# Facilitated Telemedicine as a Patient-Centered, Sociotechnical Intervention to Integrate Hepatitis C Treatment Into Opioid Treatment Programs and Overcome the Digital Divide Among Underserved Populations: Qualitative Study

**DOI:** 10.2196/68854

**Published:** 2025-07-16

**Authors:** Andrew H Talal, Arpan Dharia, Marianthi Markatou, Lawrence S Brown Jr, Kenneth E Bossert, Zakiya Grubbs, Raktim Mukhopadhyay, Boatemaa Ntiri-Reid, Elisabeth J Houtsmuller

**Affiliations:** 1 Division of Gastroenterology, Hepatology and Nutrition, Jacobs School of Medicine and Biomedical Sciences, University at Buffalo, 875 Ellicott Street, Buffalo, NY, 14203, United States, 1 716-829-3101, 1 716-854-1397; 2 Department of Biostatistics, School of Public Health and Health Professions, University at Buffalo, Buffalo, NY, United States; 3 START Treatment and Recovery Centers, Brooklyn, NY, United States; 4 Addiction Medicine and Public Health, Weill Cornell Medicine, New York, NY, United States; 5 Drug Abuse Research and Treatment, Community Action Organization of Western New York, Buffalo, NY, United States; 6 National Alliance of State and Territorial AIDS Directors, Washington, DC, United States; 7 Patient-Centered Outcomes Research Institute, Washington, DC, United States; 8 Research Triangle Institute, Research Triangle Park, NC, United States

**Keywords:** telemedicine, patient-centered, sociotechnical, hepatitis, opioid, health equity, digital divide, underserved, pragmatic.

## Abstract

**Background:**

People with opioid use disorder (OUD) have the highest rates of hepatitis C virus (HCV) infection. Despite the availability of curative HCV medication, people with OUD have limited health care access largely due to stigma. In a recent, pragmatic, randomized controlled trial (RCT), we compared a facilitated telemedicine intervention for HCV treatment integrated into opioid treatment programs (OTPs) with off-site referral. Facilitated telemedicine is bidirectional videoconferencing between a remote provider and a patient, supported by a case manager who facilitates the telemedicine encounter. The case manager schedules telemedicine visits, provides appointment reminders, and operates the digital equipment. Among 602 participants in the RCT, 90% (n=262) were cured through facilitated telemedicine and 39% (n=123) were cured through off-site referral. In this work, a multidisciplinary group of investigators, who directed the RCT, conducted a workshop, “Advancing Viral Hepatitis Screening and Treatment in Opioid Treatment Settings – Models & Resources,” at the American Association for Treatment of Opioid Dependence Conference in May 2024 to disseminate knowledge of facilitated telemedicine, including implementation considerations. We highlighted facilitated telemedicine as a patient-centered, sociotechnical, pragmatic health care delivery model for underserved populations.

**Objective:**

This study aimed to identify lessons learned to successfully overcome challenges of facilitated telemedicine implementation for HCV treatment integrated into OTPs.

**Methods:**

We partnered with the National Alliance of State and Territorial AIDS Directors in planning the workshop. The workshop consisted of 7 presentations on topics related to facilitated telemedicine implementation. The workshop was recorded and transcribed by Zoom (Zoom Communications). The transcripts served as data for the thematic analysis. The transcripts were interpreted to elucidate patterns of meanings and nuances derived from each presentation. In an iterative process, preliminary findings were compared and coalesced into themes. Verbatim quotes from the workshop were highlighted to support the themes.

**Results:**

We developed 3 themes. First, patient-centered care promotes HCV treatment for underserved populations through facilitated telemedicine. Case managers leveraged the destigmatizing environment of the OTP to build trust with patients, promoting an HCV cure through facilitated telemedicine. Second, sociotechnical approaches expand health care access for people with OUD. To be effective, facilitated telemedicine integrates 2 necessary components, a social aspect and a technical aspect. Third, facilitated telemedicine supports pragmatic research emphasizing people with OUD. Pragmatic research of facilitated telemedicine is needed to assess sustainability and scaling considerations beyond OTPs. Overall, we found that facilitated telemedicine overcame the digital divide, promoting access to digital technology, internet provision, and digital literacy.

**Conclusions:**

Facilitated telemedicine incorporates both a technical and a social component. The technical component largely addresses geographical challenges, while the social component addresses temporal (ie, care coordination) issues, promotes trust, and largely assuages patients’ concerns related to HCV treatment. The patient-centered, sociotechnical intervention can address the digital divide, thereby increasing health care access.

## Introduction

People with opioid use disorder (OUD) have the highest hepatitis C virus (HCV) incidence and prevalence due to viral transmission through injection drug use [1]. If left untreated, HCV infection can lead to cirrhosis, liver failure, and death. The development of almost ubiquitously curative, direct-acting antivirals (DAAs) for HCV in 2 to 3 months with minimal side effects has profoundly changed the therapeutic landscape since 2013 [2]. Unfortunately, however, people with OUD have had limited access to DAAs, largely due to stigma in conventional health care settings, competing priorities, unawareness of their HCV status, and service unavailability [3,4]. A recent United States nationwide study documented that only 35% of eligible individuals received DAAs from 2013 to 2022 [5]. In addition, almost 40% of people with HCV are unaware of their infection status [6]. Nonetheless, HCV treatment is a health equity priority [7]. Thus, an HCV therapeutic divide exists with insufficient penetration of highly curative DAAs among the population most in need. Furthermore, people with OUD suffer from a digital divide, defined as the unequal access to digital technology, internet provision, and gaps in digital literacy [8].

Opioid treatment programs (OTPs) provide medical and behavioral treatment for OUD in a destigmatizing, supportive, and trusting environment [9]. Integrating HCV treatment into the OTP environment can promote therapeutic interactions, patient engagement, and retention in health care. To potentially overcome the HCV therapeutic divide among people with OUD, we developed a facilitated telemedicine intervention fully integrated into OTPs using the sociotechnical system framework [10]. Recently published facilitated telemedicine results demonstrated an HCV cure in almost all treated individuals, accompanied by high patient satisfaction with health care delivery [11-13] (details of facilitated telemedicine procedures and effectiveness results are provided in Multimedia Appendix 1). Facilitated telemedicine is bidirectional videoconferencing between a remote provider and patient, supported by a case manager. The case manager established trusting relationships with patients to promote personalized HCV care approaches. The intervention can overcome geographical and temporal challenges, as well as the therapeutic and digital divides. Since facilitated telemedicine encounters are embedded in OTPs facilitated by a case manager, patients had access to digital technology, internet provision, and assistance with digital literacy. Facilitated telemedicine not only increases DAA access among people with OUD, but it can also improve service provision by OTPs.

We obtained input from a variety of stakeholders involved in the implementation and conduct of facilitated telemedicine in OTPs. We conducted a workshop entitled “Advancing Viral Hepatitis Screening and Treatment in Opioid Treatment Settings – Models & Resources.” The objective of the workshop was to disseminate successful approaches that overcame facilitated telemedicine implementation challenges. We also desired to inform workshop participants how facilitated telemedicine promotes patient-centered health care delivery for HCV treatment integrated into OTPs. We highlighted facilitated telemedicine implementation considerations that addressed the digital divide. We also highlighted specific considerations when conducting pragmatic trials, emphasizing underserved populations. We anticipate that the lessons learned could be beneficial in the expansion of facilitated telemedicine to address HCV-OUD treatment integration and health equity among people with OUD.

## Methods

### Study Design, Setting, and Sampling

We conducted a thematic analysis of the transcripts of a 3.5-hour workshop that addressed facilitated telemedicine implementation challenges. Our study’s aim was to identify facilitated telemedicine considerations that overcame implementation challenges to promote the delivery of patient-centered HCV care. The primary study outcome was the results derived from the thematic analysis. The workshop transcripts served as data for the thematic analysis.

**Figure 1.**
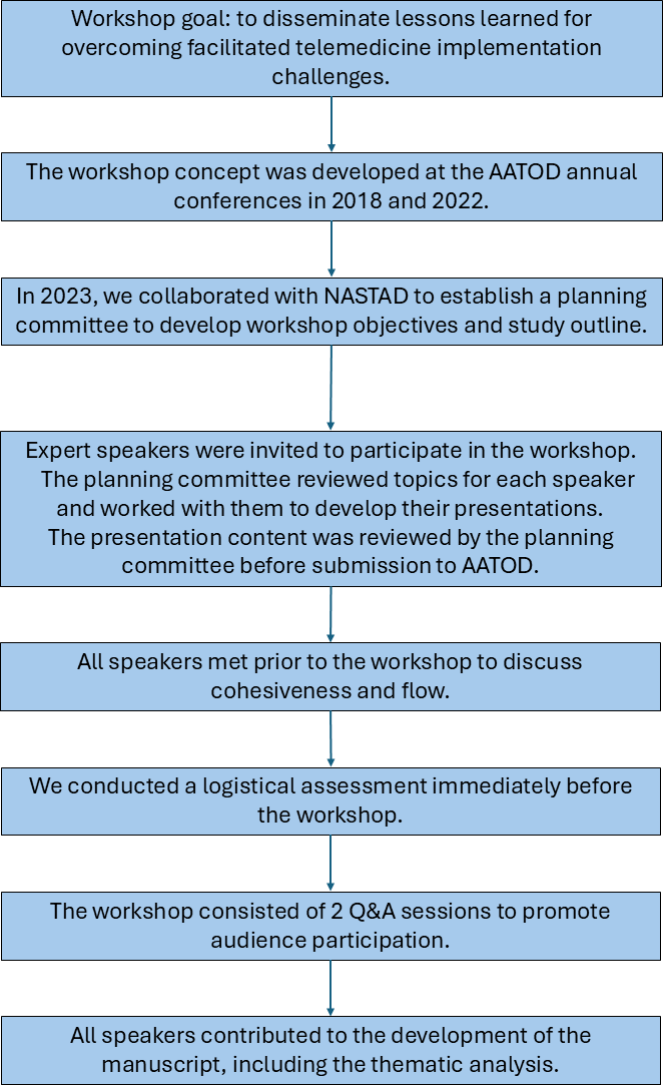
Workshop development flow diagram. The objective of the workshop was to disseminate knowledge of facilitated telemedicine for hepatitis C virus infection treatment integrated into opioid treatment programs to attendees of the 2024 American Association for the Treatment of Opioid Dependence conference. AATOD: American Association for the Treatment of Opioid Dependence; NASTAD: National Alliance of State & Territorial AIDS Directors; Q&A: question and answer.

The workshop was sponsored by the University at Buffalo (UB) and took place as a preconference workshop at the 2024 American Association for the Treatment of Opioid Dependence (AATOD) Conference [14]. The workshop occurred on May 19, 2024, in Las Vegas, Nevada. Planning for the workshop began in 2018 when 3 of the workshop speakers attended the AATOD annual meeting; the workshop planning followed an iterative process (Figure 1). The data analysis occurred in July and August 2024.

AATOD works with federal and state agencies to promote the growth and development of opioid treatment services. The association has a long history of promoting approaches to increase HCV management within OTPs, and the workshop concept originated at the 2018 annual meeting, where 3 workshop participants attended. Our workshop aligned with the AATOD conference theme of “Treating Opioid Use Disorder: So Much More Than Medication” [15]. In alignment with the conference objectives, we disseminated the outcomes of facilitated telemedicine among people with OUD. Our workshop attendees included OTP providers, OTP administrators, patient representatives, front-line staff, and state officials involved in the treatment of OUD.

### Study Intervention

The workshop activities were approved by the UB Institutional Review Board. The principal investigator (AHT) of the facilitated telemedicine trial established a collaboration with the National Alliance of State and Territorial AIDS Directors (NASTAD) in planning the workshop. The focus of the alliance is to advance the health of people impacted by HIV, viral hepatitis, and intersecting epidemics through public health advocacy, capacity building, and social justice [16]. NASTAD represents public health officials who administer HIV and viral hepatitis programs in all 50 states. NASTAD representatives participated in developing the workshop’s objectives and maintaining the relevance of the workshop through partnering with state and territorial health departments.

### Workshop Speakers

The planning committee invited workshop speakers based on their roles and a high degree of engagement in the trial. The workshop was hosted by 2 planners, 1 moderator, and 7 speakers. The planners were AHT (also a speaker) and BN-R. AHT is a physician-scientist with more than 20 years of experience in treating HCV, especially among people with OUD. He is a Professor of Medicine at UB and the principal investigator on the randomized controlled trial entitled “Integrated Hepatitis C–Opioid Use Disorder Care Through Facilitated Telemedicine A Randomized Trial” [13]. AHT serves on the New York State HCV Elimination Task Force and its HCV Guidelines Committee. BN-R is the Senior Director of Syndemic Approaches at NASTAD. She leads the integration of HIV, viral hepatitis, and harm reduction. The moderator was ZG, Senior Hepatitis Manager at NASTAD. She supports prevention and surveillance capacity building through NASTAD’s technical assistance center for viral hepatitis.

The speakers were AHT, EJH, MM, LSB, KEB, AD, and RM. EJH is a Program Director at Research Triangle Institute. Previously, she was an Associate Director at the Patient-Centered Outcomes Research Institute (PCORI) and the trial’s program officer. PCORI is an independent, nonprofit organization that funds patient-centered comparative clinical effectiveness research with emphasis on stakeholder engagement, and dissemination and implementation of successful interventions. MM is a Distinguished Professor of the State University of New York and Associate Chair for Research and Healthcare Informatics, Department of Biostatistics at UB. She was the lead biostatistician for the trial. LSB is the former chief executive officer at START Treatment & Recovery Centers, treating patients with substance use and co-occurring medical and mental health disorders. Founded in 1969, START is the oldest and largest minority-run substance use treatment program in the United States, serving over 3000 patients through 7 medication-assisted programs in New York City. KEB is the former program administrator at Drug Abuse Research and Treatment of the Community Action Organization of Western New York. Founded in 1971, Drug Abuse Research and Treatment is a free-standing community organization that provides integrated services to people with OUD throughout Western New York. KEB was also an administrative surveyor for the Commission on Accreditation of Rehabilitation Facilities. AD is a physician and the Director of Liver Services at UB. RM is a research assistant in MM’s laboratory group and a doctoral student in computational and data-enabled sciences and engineering at UB.

### Workshop Presentations Overview

The workshop was developed through planning meetings between AHT, AD, and NASTAD’s representative, BN-R. The planning committee developed an agenda with topics related to facilitated telemedicine and invited stakeholders who participated in the study by email. We used purposive sampling to select speakers who were the most appropriate to discuss each topic, as illustrated in Table 1. Furthermore, 2 individuals, representing an expert in substance use disorder and a patient-participant with lived experience, were unable to participate. These topics were represented by LSB, who has 40 years of experience in substance use treatment, and AD, who has served as a patient representative and program director during the trial. We used investigator and data source triangulation to understand when we had achieved sample saturation [17]. The workshop consisted of 7 presentations, and all invited speakers shared their presentations before the workshop. The dissemination of the trial’s findings to an AATOD audience permitted scalability opportunities to a national and international audience substantially larger than New York State, where the trial occurred.

**Table 1.** Principal workshop topics by speaker.

Topic and subtopics	Participant, name (degree)
Research	
Facilitated telemedicine	AHT (MD, MPH)
Substance use	EJH (PhD)
Social determinants of health	MM (PhD)
Data acquisition and the use of large language models	MM (PhD) and RM (MS)^a^
Perspectives and experiences	
Patients	AD (MD)
OTP^b^ leadership	LSB (MD, MPH)
OTP administration	KEB (BS)
Stakeholders	
Public health advocacy	BN-R (JD, MPH) and ZG (MPH)
Experience with OUD^c^	AATOD^d^ audience

a Speaker.

b OTP: opioid treatment program.

c OUD: opioid use disorder.

d AATOD: American Association for Treatment of Opioid Dependence.

AHT opened with an overview of the trial, “Facilitated Telemedicine for Underserved Populations: A Stepped Wedge Trial of Hepatitis C Treatment.” AHT discussed the background of HCV treatment access and health care delivery. He introduced the facilitated telemedicine model, the trial design, primary and secondary outcomes, and the trial’s strengths, limitations, and lessons learned.

EJH followed with “Funding Projects in People with OUD.” EJH explained that comparative effectiveness research funded by PCORI must be patient-centered, stakeholder-driven, and pragmatic, and that the facilitated telemedicine trial met all 3 criteria. She described the recent increase in patient-centered approaches for people with OUD. EJH highlighted several patient-centered parts of the final rule of the Substance Abuse and Mental Health Services Administration that was released in April 2024 [18]. The Substance Abuse and Mental Health Services Administration final rule expands treatment access by permitting telehealth evaluation, replacing stringent dosing requirements with clinical judgment, removing daily visit requirements, and meeting OUD diagnostic criteria. EJH cautioned that critical barriers remain, such as internet access in rural and underserved areas and for those with limited digital literacy.

MM presented on “Improvements in Substance Use and Social Determinants of Health (SDOH).” She discussed how substance use and SDOH can be evaluated as predictors for HCV treatment initiation and cure. MM explained that integrated HCV and OUD treatment resulted in improvements in substance use, education, employment, and criminal justice status. However, since treatment for HCV and OUD is not well-integrated into health care systems, managing these conditions will require addressing social and structural inequalities.

LSB discussed “Drivers for HCV-Related Telemedicine Research.” He described the critical relationship between public policy and clinical care as he was originally from a neighborhood in which substance use was, and unfortunately still is, part of the landscape. LSB provided an overview of START Treatment and Recovery Centers in New York City. He explained that facilitated telemedicine aligned very closely with START’s corporate philosophy of participating in medical and behavioral research and advocacy to address unmet patient needs.

KEB presented “The Evolution of Incorporating Facilitated Telemedicine in an Opioid Treatment Program: An Administrator’s Perspective.” KEB defined the notions of the “phenomenon of the OTP” and “full-service garage.” He described the “phenomenon of the OTP” as a natural incubator of trust because the OTP provided structure, purpose, a safe environment, and socialization to people with OUD. The “full-service garage” implies that the OTP offers a panoply of health care and social services beyond medication-assisted treatment. KEB emphasized the interactions of facilitated telemedicine with both concepts.

AD discussed “Facilitated Telemedicine: Overcoming Challenges for Hepatitis C Treatment.” He presented patient testimonials of facilitated telemedicine derived from qualitative interviews [3]. Patients were enthusiastic about health care delivery through facilitated telemedicine, and an HCV cure promoted self-confidence and improved whole health. AD also reviewed the CREATE (Culture, Respect, Educate, Advantage, Trust, Endorse) framework, an engagement approach for facilitated telemedicine [9].

RM concluded the workshop with “From Words to Wellness: Large Language Models Applied to the Identification of SDOH.” He explained that SDOH data are limited in people with HCV, and he described approaches used to derive SDOH data from trial participants [19]. RM concluded his presentation with a discussion of issues of quality and the application of models for the identification of SDOH derived from people with OUD.

Once the speakers had agreed to present at the workshop, they worked iteratively with the planning committee to obtain feedback on the presentations. When the presentations were finalized, each speaker uploaded the presentation to the AATOD website. Workshop speakers met in person before the workshop to review the presentations’ content and to promote cohesiveness and flow. Speakers reviewed logistical considerations immediately before the workshop, including timing, recording, and moderation by NASTAD staff. Furthermore, 2 question-and-answer sessions were held during the workshop that were moderated by ZG. During the introductory remarks, the moderator discussed the objectives of the conference, the context of HCV with a special emphasis on the importance of HCV elimination, and specific areas of discussion for the audience. To minimize recollection bias, the question-and-answer sessions occurred immediately after the workshop presentations. As the workshop presenters had extensive experience working collaboratively to achieve the trial’s goals, respondent bias was minimized. On the other hand, however, the moderator was not previously known to the workshop presenters and represented a public health advocacy perspective, which encouraged discussion of a diverse array of perspectives. To ensure question comprehension, the moderator repeated the question before eliciting a response. The moderator also sought responses from multiple workshop presenters as appropriate. After completion of the workshop, the planning committee met to discuss topics for manuscript development. The primary focus of the planning committee was the thematic analysis of the workshop presentation transcripts.

### Thematic Analysis

The 3.5-hour workshop was recorded and transcribed by Zoom (Zoom Communications). All workshop speakers disseminated their successful approaches that overcame facilitated telemedicine implementation challenges. After the final set of transcripts was obtained, they were reviewed for completeness independently by 2 investigators (AHT and AD), with inconsistencies verified by listening to the recordings. While the transcripts did have identifiable quotations, those that were used in the analysis were deidentified. The workshop transcripts served as the raw data and the primary source for the thematic analysis. Since all workshop presenters had worked collaboratively for 8 years on evidence generation as part of the trial, the workshop adhered to the principle of investigator triangulation. The development of multiple manuscripts covering a variety of different perspectives from the trial adhered to the use of data triangulation [17].

Using a constructivist paradigm, we performed a thematic analysis to identify facilitated telemedicine considerations that overcame implementation challenges and delivered patient-centered care. In total, 2 investigators, AD and AHT, followed the 6-step framework for thematic analysis described by Braun and Clarke [20,21]. AD and AHT have published several qualitative studies in Q1 journals [3,9,22-27] and are well-versed in the approaches to minimize bias in qualitative research. They became familiar with the data by independently reviewing the transcripts for accuracy and formatting. They independently and inductively coded the transcript data based on its relevance to facilitated telemedicine considerations that overcame implementation challenges and delivered patient-centered care. Subsequently, they independently grouped the initial codes into general categories. In a recursive process, they discussed code generation to identify and interpret repeated patterns of meaning and nuances derived from each presentation. They met frequently to discuss these patterns of meaning until a consensus was reached and lower-level themes were developed with supporting quotations [28]. In the case of disagreements in the textual interpretations, they reviewed the transcripts until a consensus was reached.

Once a draft of concepts had been derived and agreed upon, the lower-level themes were discussed and reviewed with all coauthors in a series of meetings. Using a consensus and iterative approach, lower-level themes were defined and evolved into higher-level themes. Verbatim quotes from the workshop presentations were highlighted to support the themes. All coauthors reviewed the final themes and supporting quotes for agreement. All coauthors were active participants in the data-driven thematic analysis. We acknowledge that the experiences and interactions of the coauthors may have influenced the thematic analysis in this constructivist paradigm. As a concise report of qualitative analytical methods used, the Consolidated Criteria for Reporting Qualitative Research (COREQ) checklist is provided (Multimedia Appendix 1).

Because of time constraints, several aspects related to care delivery through facilitated telemedicine could not be addressed at the workshop and are discussed in detail in separate papers. Specifically, the aspects that led to successful case management are discussed in a study by Talal et al [27]. The considerations for effective case conferencing at the patient and system levels are discussed in [22,29], respectively. Finally, the issues related to care delivery in studies by Ventuneac et al [23], Dickerson et al [24], and Talal et al [unpublished data, 2025] describe the considerations for scaling and maintaining facilitated telemedicine, the characteristics that led to full integration of facilitated telemedicine into OTP workflows,, and implementation characteristics required for effective treatment outcomes and high levels of patient satisfaction, respectively. In addition, the issues of data ownership, sharing, and privacy were not discussed at the conference, as these topics had been outlined in the trial protocol approved by the institutional review board.

### Ethical Considerations

The study was approved by the Institutional Review Board at UB (MODCR00009273) (Multimedia Appendix 2). All workshop speakers consented to participate. All quotations were deidentified. Confidentiality concerns prohibit us from sharing data transcripts. We provided reimbursement for travel, lodging, and meals. Artificial intelligence was not used in any portion of this study.

## Results

**Figure 2.**
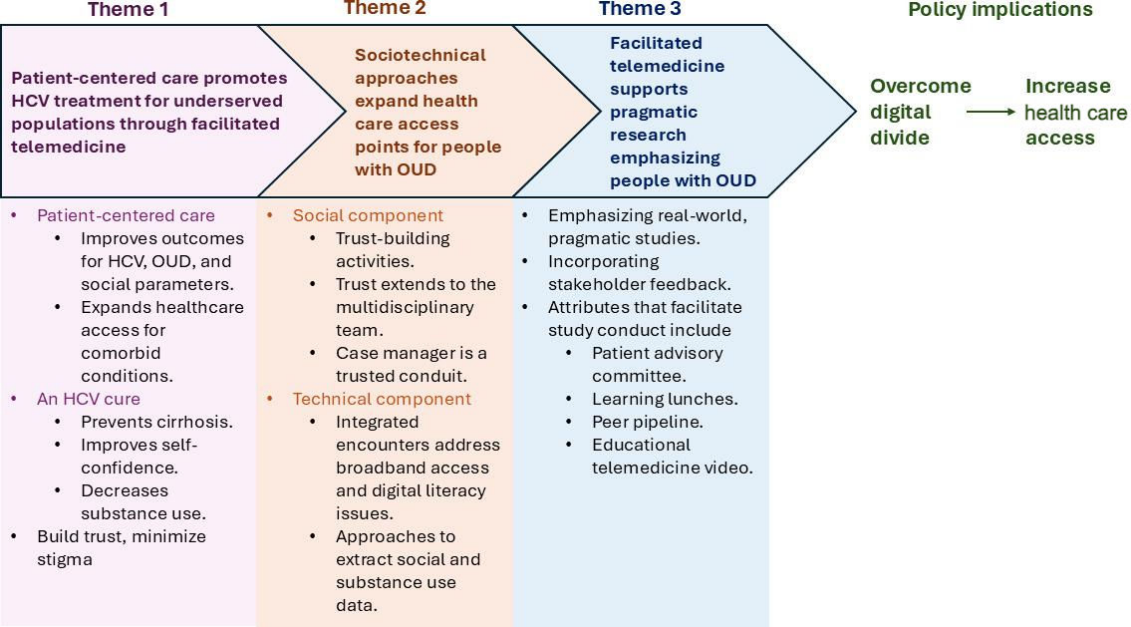
Three themes and important attributes identified through thematic analysis of workshop presentations at the 2024 American Association for Treatment of Opioid Dependence Conference. The objective of the workshop was to disseminate knowledge of facilitated telemedicine for hepatitis C virus treatment. We highlighted facilitated telemedicine as a patient-centered, sociotechnical, pragmatic health care delivery model for underserved populations. We found that facilitated telemedicine integrated into opioid treatment programs overcame the digital divide. HCV: hepatitis C virus; OUD: opioid use disorder.

We developed 3 themes from the textual analysis of the workshop presentations (Figure 2).

### Theme 1: Patient-Centered Care Promotes Hepatitis C Treatment for Underserved Populations Through Facilitated Telemedicine

Patient-centered care for HCV and OUD promotes improvements not only in medical conditions but also in social factors. Patient-centered care emphasizes promoting individual health care choices and empowering patients to actively participate in health care decision-making [30] and is delivered by a multidisciplinary team.

*[The] approach is to treat the whole person, and not just their substance use disorder… They have co-occurring alcohol use disorder… HIV, hepatitis C, diabetes, hypertension, and psychiatric disorders*.

*[HCV and OUD treatment] promotes improvements in substance use and social functioning, [specifically] education, employment, and involvement with the legal system*.

*The patients were actually helped to go back to school and complete whatever degrees they had [to complete]*.

Consistent with the strong potential benefits of integrated OUD and HCV care, an OTP administrator cited his desire for participation in facilitated telemedicine as the “full-service garage” concept, providing essential medical care within the OTP for a large repertoire of comorbidities.

*The full-service garage sentiment is the leverage of the trust equity and utilizing facilitated telemedicine provided a hub and spoke… If OTPs reach out to community providers, it becomes the home base for consumers and facilitated telemedicine becomes the opportunity to engage in essential services without having to leave the clinic*.

The value of integrated care was illustrated through obtaining an HCV cure, which represented a major achievement for people with OUD.

*For patients that successfully completed [HCV treatment], it was the first major win for them. They realized that they had an active role in their recovery… Having a personal win that they could own was profound and had long term effects*.

An HCV cure removed the risk of cirrhosis and improved substance use.

*He [trial PI] would actually use ‘cure’, and that’s a 4-letter word we don't use in the [methadone] program*.

*The message to take home is that once the patient initiates the treatment, you have a high probability that the patient is actually going to stick with it and obtain an HCV cure*.

*Patients valued the HCV cure because it promoted self-confidence, and it enabled them to improve their health and lives*.

Among cured participants, we observed significant decreases in substance use, as assessed through the drug abuse screening test [31], with minimal HCV reinfections [13]. An HCV cure enabled the treatment team to reinforce adherence to OUD treatment.

*When the patients progress through the study, the counselors and the treatment team will often utilize that [HCV cure] as a pivot with the patient… It was a wonderful tool that the treatment team used to support a patient’s recovery*.

To engage people with OUD in research, we pursued approaches to build trust, such as integrating facilitated telemedicine encounters into the supportive, destigmatizing, and trusting environment of the OTP [32,33]. The foundation of facilitated telemedicine is patient-centeredness. Patients endorsed facilitated telemedicine because they realized the profound effects of an HCV cure.

*The OTP became an extension of their family… A place of trust*.

People with OUD frequently encounter stigma [34] and feel ostracized in conventional health care settings. Stigma is a major impediment to health care access.

*They describe themselves as addicts, dirty or clean. Patients blame themselves for contracting a lethal disease [i.e., HCV]. They face stigma in conventional healthcare settings outside of the OTP*.

*We had tried multiple attempts to refer out [for HCV]… It was really met dismally. For some, it was logistics. They had a hard time getting to the other location. But for a lot of them, it was stigma*.

The approaches we used to maintain patient-centeredness are described in Multimedia Appendix 1. The growth of patient-centeredness in the treatment of substance use disorders has been gaining considerable momentum [35].

*The vision of the agency [OTP] is dedicated to transforming the perception of addiction and behavioral health disorders by bringing dignity and respect to the lives, families, and communities we serve*.

### Theme 2: Sociotechnical Approaches Expand Health Care Access for People With OUD

The sociotechnical system framework, which we used to evaluate the facilitated telemedicine intervention, is specifically designed to address challenges in telemedicine implementation in adaptive, complex health care systems, such as OTPs [29]. The facilitation aspect is the social component of facilitated telemedicine and was critical when designing digital health solutions for people with OUD.

*[We had] to think about cultural and trust issues that may diminish its [telemedicine’s] acceptability*.

*Case managers were communicating information about the study, enrollment, retention, and all things hepatitis C beyond the study*.

*[We had to] gain trust in the OTP to participate in patient-centered telemedicine encounters*.

An OTP leader explained the benefits of facilitated telemedicine and his reason for pursuing the intervention. He described the 4 pillars that guided his leadership strategy.

*Our corporate philosophy is recognizing and overcoming OTP-related challenges like continuing relationships and addressing patient needs*.

*Patient care, education, advocacy, and research... Telemedicine offers a number of opportunities to enhance value-based clinical care*.

The social structure within the OTP promoted trial enrollment and retention. Trust extended to the multidisciplinary team.

*Early on, the treatment team did a lot of priming of the pump regarding recruitment… I thought it was important to provide education about the study to everyone because the trust equity that the patients have certainly was to the doctors, nurses, and counselors. But sometimes, their strongest relationships were with security staff or a business office person that they saw every day*.

During the question-and-answer periods, several audience members asked about the requirements to replicate the facilitated telemedicine intervention, including social parameters.

*Remember the eligibility requirements [for the trial]. You had to be in [OUD] treatment for some period of time [6 months] so that means that there was a relationship that was developed between that patient and their counselor, as well as the employees of our program, who are related to the study. There was a relationship that had started to flourish at the time prior to [trial] enrollment*.

The other aspect of facilitated telemedicine is the technical component. We mitigated digital divide issues, such as technology access, digital health literacy, and broadband access, by fully embedding telemedicine encounters into the OTP. During the COVID-19 pandemic and lockdown, facilitated telemedicine became an approach to maintain patient engagement.

*If one is thinking about adding a telehealth solution, one needs to consider the digital divide. Specific concerns would be access to the internet, digital health literacy, and medical-behavioral integration with virtual care*.

*COVID-19 tethered the consumer away from the clinic… Telemedicine became the umbilicus; it brought the patient back*.

We developed workflows to extract patient-level data on social factors to support facilitated telemedicine implementation as well as for identifying social factors that might be associated with HCV treatment uptake and cure. These tools enabled cross-domain collaboration as well as played crucial roles in ensuring reproducibility and in optimizing clinical and social data acquisition and their integration [19]. An interest in the use of artificial intelligence as a component of OTP operations was discussed during the question-and-answer period.

*Recently, large language models have shown the capacity to process vast amounts of data to generate insights... We are interested in how these models can aid in the identification of social factors… to explore relationships between social factors and pursuit of HCV [treatment]*.

These approaches are permissive for the direct inclusion of social factors and substance use variables in the patient’s treatment plan.

*[The analytical goal is] to emphasize social factors that can be integrated into the treatment approach*.

Facilitated telemedicine successfully manages the digital divide by addressing both social and technical challenges faced by people with OUD, as substantiated in Table 2.

**Table 2.** Sociotechnical system framework for facilitated telemedicine supported by workshop examples. Table adopted from study by Talal et al [29].

Sociotechnical system framework constructs	Definition	Workshop examples	Sociotechnical component
People	Clinicians, patients, administrators, researchers, or software developers.	“Case managers were communicating information about the study, enrollment, retention, and all things hepatitis C beyond the study.”“[We had to] gain trust in the OTP^a^ to participate in patient-centered telemedicine encounters.”	Social
Internal organizational features	Internal organizational policies, procedures, and institutional culture	“Our corporate philosophy is recognizing and overcoming OTP-related challenges like continuing relationships and addressing patient needs... Telemedicine offers a number of opportunities to enhance value-based clinical care.”	Social
Hardware and software computing infrastructure	Technical requirements, (eg, hardware, equipment, devices, broadband)	“If one is thinking about adding a telehealth solution, one needs to consider the digital divide.”	Technical
Human-computer interface	Comfort level and overall user-friendliness of the interface	“COVID-19 tethered the consumer away from the clinic… Telemedicine became the umbilicus; it brought the patient back.”	Sociotechnical
Workflow and communication	Logistics surrounding the proper functioning of the system and ensuring cohesive communication	"I thought it was important to provide education about the study to everyone because the trust equity that the patients have certainly was to the doctors, nurses, and counselors.”	Sociotechnical

a OTP: opioid treatment program.

### Theme 3: Facilitated Telemedicine Supports Pragmatic Research Emphasizing People With OUD

The tremendous efficacy of DAAs promoted support for pragmatic effectiveness studies for HCV. Simultaneously, the trial sponsor emphasized pragmatism in research.

*There was real interest in hepatitis C treatment and that opened the channels for funding OUD studies*.

*The studies had to be done in the real world, and they had to include the people who are implementing the treatment and the patient-centered aspects of telehealth*.

Simultaneously, however, various stakeholders voiced concerns about engaging people with OUD in research.

*The concerns regarding funding studies in people with OUD very much reflected the ones that payers and providers had… Grave concerns about treating this particular population because of its reputation for being unstable, that they would not complete treatment, would get reinfected, or would disappear*.

Historically, clinical research was largely focused on academia and was considered complete when the study results were published.

*Studies were developed by researchers and then evaluated by other researchers… The problem with academic research was that if there was nobody who wanted to implement what was found effective, then nothing happened with it*.

Pragmatic studies achieve their full potential when they have long-lasting impact. In our case, 4 of 8 sites have continued to use facilitated telemedicine for evaluation of complex cases.

We used a variety of techniques, including the patient advisory committee, peer pipeline, and learning lunches, to understand and address facilitated telemedicine implementation challenges within the OTP. The patient advisory committee facilitated participant understanding of facilitated telemedicine through recommendations to augment digital literacy. For example, we developed an educational video explaining facilitated telemedicine that we showed to participants during recruitment discussions.

*We had a patient advisory committee that… formed a very cohesive bond and served as a mechanism for discussion and feedback on study-related interventions even before we implemented them*.

Alongside the patient advisory committee, there was a peer pipeline where patients had the opportunity to endorse trial participation to their peers.

*We had a peer pipeline… Patients spoke to other patients that promoted subsequent engagement and maintained retention*.

*They [patients] became the strongest advocates for enrollment into the study*.

We discovered that learning lunches were a particularly important mechanism to provide staff education, to facilitate OTP staff and research team interactions, and to receive feedback for potential course correction of trial procedures [9].

*Learning lunches were open for the entire multidisciplinary team… Patient care providers, billing department and our security staff because many of them were seeing the patients every day… Having that opportunity to work through some of those knots was very instrumental to the [trial’s] success*.

In addition to OTP leadership, patients, and trial sponsors, stakeholder engagement extended to the clinical and nonclinical staff. It was important to regularly communicate with OTP staff as they managed OTP workflows. Ongoing stakeholder engagement, combined with program funding, was synergistic toward facilitated telemedicine implementation, as explained by OTP leadership.

*I’ve come to appreciate the need to have continuing relationships… Those relationships are not just research, but they are also economic. When you can get funding from a research institute to support what you do, it makes a big difference in day-to-day operations*.

HCV treatment access is a health equity priority [7]. Therefore, funding and policy should be evidence-based. Future research funding should focus on the scaling and sustainability of facilitated telemedicine to promote health care access.

*Public policy is not always driven by science or clinical practice*.

*What we need are some creative solutions to the access problem. Telehealth takes care of part of it, but we also need to think about where telehealth doesn’t work… We still have the great digital divide. There are people who have no access to the internet... You have to have some familiarity with the technology… Not everybody has digital literacy*.

## Discussion

### Principal Results

Facilitated telemedicine, through its transcendence of geographical and temporal boundaries, resulted in remarkably high HCV cure rates when integrated into 12 OTPs throughout New York State. Implementing the intervention, however, required convincing stakeholders of the benefits of facilitated telemedicine. We conducted a pragmatic randomized controlled trial in people with OUD that satisfactorily addressed the digital divide in an underserved population and that successfully implemented a novel health care delivery modality using a complex design. We found that open and honest stakeholder discussions were usually sufficient to overcome initial resistance regarding integrating facilitated telemedicine into OTPs. Conducting clinical research in OTPs and recruiting people with OUD were novel concepts when the trial was proposed because neither typically participated in research. An initial challenge was convincing stakeholders to endorse a trial directed toward the setting and population. An additional challenge was whether true uncertainty (ie, equipoise) existed between the interventions being tested in the clinical trial. Since it was difficult to establish whether true uncertainty existed, we used the stepped wedge design [36]. The concept of using the stepped wedge design was also considered a potential complexity given its stringent enrollment and timing requirements. Patient engagement in a trusting and destigmatizing environment by staff, who had the patients’ best interests as their priority, mitigated several of these concerns. From these perspectives, the OTP environment enabled us to fully integrate HCV treatment through facilitated telemedicine into its workflows. After the trial’s conclusion, we have continued facilitated telemedicine for evaluation of complex cases in 4 sites. In 8 sites, facilitated telemedicine served as a bridge to onsite HCV treatment by OTP staff. From our investigation, we have learned that facilitated telemedicine can increase health care access using a value-based care approach.

### Digital Divide

The digital divide remains a challenge to the scalability of facilitated telemedicine. The digital divide refers to the unequal access to digital technology, internet provision, and digital literacy that worsens inequality around access to information and resources [8]. In the design of facilitated telemedicine, we considered social and technical aspects, which were facilitated by a case manager. During telemedicine encounters and videoconferencing for patient advisory committee meetings, the case manager facilitated equipment operation. The case manager also addressed patients’ questions and concerns and was an information conduit for any HCV-related issues. The importance of considering the social aspects of digital connectivity was demonstrated most poignantly during the COVID-19 pandemic and lockdown, when patient advisory committee meetings occurred weekly (instead of quarterly) through videoconferencing. The committee meetings became an extremely important approach to maintain patient engagement. In resource-limited settings, OTP staff can assume the role of HCV champion. Therefore, facilitated telemedicine has the potential to reach underserved populations (ie, migrants, incarcerated individuals, and the homeless) and address the digital divide through specific approaches as illustrated (Table 3). The importance of the social aspects when increasing digital health care access to underserved populations has been investigated among people who use drugs in Athens, Greece [26,37]. Participants emphasized the importance of trust-building, including several suggestions to enhance trust through digital means, during focus group discussions. More importantly, social interaction and relationship building (ie, empathy, trust, support, and transparency) contributed to the patient-centeredness of facilitated telemedicine. Therefore, telemedicine without the “facilitation” aspect would be devoid of the social component that is crucial for high-quality, patient-centered care [24], especially for populations that often experience stigma from various sources. In this context, the social component was largely delivered by the case managers who facilitated the telemedicine encounters, educated patients, and served as patient advocates. Other facilitated telemedicine approaches have employed staff or peers to facilitate the telemedicine encounters [38-40]. The sociotechnical approach also has the potential to present the OTP as a “full-service garage” that can address OUD-related infectious diseases, mental health issues, common comorbidities, and social factors. The novelty of facilitated telemedicine remains the integration of the social and technical aspects for underserved populations.

**Table 3.** Lessons learned through the implementation of facilitated telemedicine to overcome the digital divide.

Topic	Lessons learned and recommendations
Medical	Integrate HCV^a^ and opioid use disorder treatment Address medical comorbidities
Social	Minimize stigma against underserved populations Maintain patient-centeredness Promote trust Address competing priorities Provide patient education Employ case manager as patient advocate and patient navigator
Technical	Facilitate telemedicine encounters Promote access to technology Ensure sufficient broadband strength Negate digital literacy challenges
Lessons for pragmatic research	Promote learning lunches and establish patient advisory committees Reinforce the importance of HCV cure by OTP^b^ staff Disseminate information through peer pipeline Engage stakeholders Emphasize underserved population Consider telemedicine sustainability through reimbursement

a HCV: hepatitis C virus.

b OTP: opioid treatment program.

Pragmatic research related to facilitated telemedicine implementation, conventionally conducted as explanatory clinical trials, sought to evaluate an intervention while controlling for multiple factors [41]. Within the past decade, increasing value has been placed on pragmatic research, including stakeholder engagement, implementation evaluation in real-world settings, and sustainability of effective interventions following study completion (Table 4).

**Table 4.** Comparison between explanatory and pragmatic research related to facilitated telemedicine implementation. Table adopted from studies by Le-Rademacher et al [41] and Krist et al [42].

Domain	Explanatory research	Pragmatic research	Facilitated telemedicine examples
Impact	To gain scientific knowledge	To make real-world changes	Facilitated telemedicine overcoming digital divide
Patient relevance	Patients are typically solely research participants	Usually patient-centered research matters to patients and caregivers; patient involvement in all study phases	Patients readily view the advantages of facilitated telemedicine
Stakeholder involvement	Largely investigator focused	Multiple stakeholders engaged in all study phases	OTP^a^ staff referred patients for facilitated telemedicine
Research design	Extensive exclusion and inclusion criteria often lead to homogenous populations and settings	Real-world settings, populations, contextual assessments; usually parallel or stepped-wedge design usually with a high degree of heterogeneity.	Research in opioid treatment programs with usual workflows, development of a statewide telemedicine network.
Measures	Validated measures that minimize bias; focus on internal consistency	Practical to real-world and low-resource settings; matter to patients	HCV^b^ cure, satisfaction with healthcare delivery through telemedicine
Costs	Infrequently collected	Assess cost of intervention	Telemedicine reimbursement
Data sources	Data collection from patients as part of the trial.	Data collected at patient and site level; can use existing datasets (ie, health records, administrative data, and patient lists)	Remote access to electronic health records, case managers faxing laboratory reports
Analyses	Can be per protocol	Intention-to-treat, relevant to multiple stakeholders; goal is understanding implementation process	Patient satisfaction with health care delivery through facilitated telemedicine.
Intervention adherence and follow-up requirements	Close monitoring and follow-up	Can be as usual care	Case managers ensured adherence to study and clinical procedures.
Sustainability	Emphasis on efficacy under ideal circumstances	Emphasis on effectiveness in real-world settings	HCV management of complex cases through telemedicine at 4 sites, onsite HCV treatment at 8 sites after study completion.

a OTP: opioid treatment program.

b HCV: hepatitis C virus.

Health care delivery approaches, such as facilitated telemedicine, are most effectively investigated through pragmatic as opposed to explanatory trials. The typical goal of explanatory research is to evaluate the efficacy of an intervention in a controlled setting, whereas pragmatic research evaluates its effectiveness in routine clinical practice settings to maximize applicability, generalizability, and transferability [43]. Explanatory research typically measures clinical symptoms or biological markers, whereas pragmatic research focuses on multiple outcomes, many of which are patient-centered. Pragmatic research emphasizes stakeholder involvement, real-world settings and populations, and outcomes that are relevant to patients and stakeholders. Therefore, data collection is typically more laborious and is required from patients, sites, and communities [41]. In addition, since sites where pragmatic studies are conducted are often part of a health care system, they typically share personnel, policies, and resources. Thus, the assumption that study sites are independent of each other, typically observed in explanatory studies, may be violated in pragmatic trials. Proper analysis of pragmatic trial data may require complex statistical models that can account for correlated responses between clusters. In the trial, we were fortunate to have a negligible correlation between our study sites [36]. Furthermore, we developed a comprehensive dataset that permitted the analysis of substance use and social factors in relation to HCV treatment initiation and cure [44].

### Strengths and Limitations

Strengths of the workshop include multidisciplinary speakers who directed a multiyear trial of facilitated telemedicine. The presentations have been substantiated with extensive staff and patient interviews [3,22-24]. Another strength was partnering with national organizations (ie, AATOD, NASTAD, and PCORI) that represent patients with HCV and OUD and their caregivers. The workshop provided the opportunity to disseminate the findings of the trial to a national audience that specializes in OUD care. A minor limitation is the nonrecognition of all spoken words by the recording and transcription program; less than approximately 1% of spoken words were not picked up by the recording. In addition, a patient-participant was unable to attend the workshop. Fortunately, the patient’s voice was communicated through a presentation about patient perspectives derived from a recent publication [3]. Given that the age of 18 years or older was a trial inclusion criterion, we are unable to generalize the findings from this investigation to younger-aged individuals.

### Conclusions

We developed facilitated telemedicine as a patient-centered intervention designed to expand DAA access among people with OUD. Because people with OUD may have difficulty crossing the digital divide, we used facilitated telemedicine to engage and retain the population in HCV care. A multidisciplinary group of investigators hosted a workshop to disseminate knowledge of facilitated telemedicine for HCV care integrated into OTPs. From the workshop presentations, we identified 3 themes. First, patient-centered care promotes HCV treatment for underserved populations through facilitated telemedicine (theme 1). Second, sociotechnical approaches expand health care access for people with OUD (theme 2). While the technical aspect of facilitated telemedicine was conducive to overcoming geographical and temporal obstacles, we had to consider the social aspect in the delivery of HCV care through digital approaches to people with OUD. These were primarily addressed by integrating HCV telemedicine encounters into the destigmatizing, supportive, and trusting OTP environment and by facilitation through a case manager.

Third, facilitated telemedicine supports pragmatic research emphasizing people with OUD (theme 3). Initial resistance of OTP leadership and frontline staff regarding facilitated telemedicine was effectively mitigated through engagement of a patient advisory committee, a peer pipeline operating within the OTP, and learning lunches. These approaches facilitated participant and OTP staff input on approaches to improve facilitated telemedicine. Addressing the digital divide requires developing patient-centered, sociotechnical innovations for health care delivery and equity directed toward underserved populations.

## Supplementary material

10.2196/68854 Multimedia Appendix 1 Supplementary materials.

10.2196/68854 Multimedia Appendix 2 Study protocol.

## Abbreviations

AATOD: American Association for the Treatment of Opioid Dependence
COREQ: Consolidated Criteria for Reporting Qualitative Research
DAA: direct-acting antiviral
HCV: hepatitis C virus
NASTAD: National Alliance of State and Territorial AIDS Directors
OTP: opioid treatment program
OUD: opioid use disorder
PCORI: Patient-Centered Outcomes Research Institute
RCT: randomized controlled trial
SDOH: social determinants of health
UB: University at Buffalo
