# Supplementary material for: Facilitated Telemedicine as a Patient-Centered, Sociotechnical Intervention to Integrate Hepatitis C Treatment Into Opioid Treatment Programs and Overcome the Digital Divide Among Underserved Populations: Qualitative Study
Source: JMIR Public Health Surveill. 2025 Jul 16;11:e68854. doi: 10.2196/68854 (PMC12286564; doi:10.2196/68854)
Supplement: Multimedia Appendix 1 [file publichealth-v11-e68854-s001.docx]

# Multimedia Appendix 1

Contents

[Multimedia Appendix 1 1](#_Toc195870719)

[Description of the randomized controlled trial procedures 1](#_Toc195870720)

[Facilitated telemedicine effectiveness 1](#_Toc195870721)

[Patient-centeredness 1](#_Toc195870722)

[COREQ Checklist 3](#_Toc195870723)

[Supplemental references 10](#_Toc195870724)

### Description of the randomized controlled trial procedures

The Patient-Centered Outcomes Research Institute (PCORI) supported a 7-year, multisite, nonblinded, pragmatic, stepped wedge, randomized controlled trial to compare facilitated telemedicine integrated into 12 OTPs throughout New York State (NYS) to offsite referral for HCV treatment [1]. The trial utilized the stepped wedge design that required high-level collaboration between the research team, clinic staff and administrators, and data and statistical science experts. The multidimensionality of facilitated telemedicine involved multiple stakeholders without placing undue burden on OTP workflows. In the facilitated telemedicine intervention, the case manager is an advocate, educator, and facilitator of telemedicine encounters [2]. A total of four protocol-stipulated telemedicine encounters occurred during the trial. At the initial encounter, the case manager and OTP healthcare provider were in the room with the patient in the OTP; the hepatitis specialist joined remotely through telemedicine.

### Facilitated telemedicine effectiveness

We utilized the stepped wedge design with site randomization. The trial was conducted over four 9-month periods and trial design details are provided in [1]. All 12 sites initially participated in offsite referral. After each 9-month period, 4 randomly selected sites transferred to facilitated telemedicine. By the fourth 9-month period, all 12 sites participated in facilitated telemedicine. We enrolled 602 participants from March 1, 2017, to February 29, 2020. In facilitated telemedicine, 90.3% of participants achieved an HCV cure, compared to 39.4% in offsite referral [3]. We also observed that substance use decreased significantly among cured participants, and there were 2.5 HCV reinfections per 100 person-years [3]. Participants in both trial arms rated healthcare delivery satisfaction as high or very high using the Patient Satisfaction Questionnaire [4]. We also created a patient advisory committee that met quarterly through videoconferencing to promote digital literacy and patient engagement.

### Patient-centeredness

We established patient-centeredness through an initial pilot study in which 93% of participants achieved an SVR [11] and where we also assessed OTP patients’ reactions to facilitated telemedicine [10]. The pilot study offered an opportunity to develop approaches to improve empathy and patient-provider trust through telemedicine. These approaches, such as a warm handoff between the OTP provider and the remote hepatitis specialist, became cornerstones of the facilitated telemedicine encounters during the conduct of the randomized controlled trial. Through patient-participant interviews, we learned the value of communication between OTP staff and research team members, the importance of gaining trust during telemedicine encounters, and of visualizing the advantages of virtually integrated HCV treatment [16]. We also learned that an HCV cure promotes interest in whole-health [3]. Similarly, OTP staff interviews revealed the premium they placed on an HCV cure [17] and how they viewed the potential of facilitated telemedicine to address other pressing health issues in this population [18]. OTP staff revealed that telemedicine had not previously been used in the sites, although their experiences during the study was extremely helpful when all medical appointments shifted to virtual during the COVID-19 lockdown. Patient-centeredness was reinforced by the engagement of multiple stakeholders focusing on achieving an HCV cure amongst people with OUD [9].

OTPs in NYS are under the regulatory oversight of the NYS Office of Addiction Services and Supports that certifies and monitors a network of prevention, treatment, and recovery providers to ensure that OTPs operate in strict compliance with NYS regulations. The collaboration with the state office also enabled us to obtain data on substance use and social factors prior to HCV treatment [19]. We were able to use these data to evaluate the additive effect of HCV treatment initiation on sustaining and enhancing improvements in substance use and social factors that commenced with methadone initiation [20].

### COREQ Checklist

| **Characteristic** | **Guide questions/description** | **Manuscript page number** | **Description or relevant text from manuscript.** |
| --- | --- | --- | --- |
| **Domain 1: Research team and reflexivity** | | | |
| *Personal Characteristics* | | | |
| 1. Interviewer/   facilitator | Which author/s conducted the interview or focus group? | Manuscript page 5 | The workshop was hosted by 2 planners, 1 moderator, and 7 speakers. The planners were Andrew H. Talal and Boatemaa Ntiri-Reid. The moderator was Zakiya Grubbs. The speakers were Andrew H. Talal, Elisabeth J. Houtsmuller, Marianthi Markatou, Lawrence S. Brown, Kenneth E. Bossert, Arpan Dharia, and Raktim Mukhopadhyay. |
| 2. Credentials | What were the researcher’s credentials? E.g. PhD, MD | Manuscript page 5-6 | Andrew H. Talal, MD, MPH  Arpan Dharia, MD  Marianthi Markatou, PhD  Lawrence S. Brown Jr., MD, MPH  Kenneth E. Bossert, BS, CASAC  Zakiya Grubbs, MPH  Raktim Mukhopadhyay, MS  Boatemaa Ntiri-Reid, JD, MPH  Elisabeth J. Houtsmuller, PhD |
| 3. Occupation | What was their occupation at the time of the study? | Manuscript page 5-6 | Dr. Talal is a Professor of Medicine with more than 20 years of experience in treating hepatitis C virus (HCV). Ms. Ntiri-Reid is the Senior Director of Syndemic Approaches at NASTAD. Zakiya Grubbs is the Senior Hepatitis Manager at NASTAD. Dr. Houtsmuller is a Program Director at Research Triangle Institute and a former Associate Director at PCORI. Dr. Markatou is a Distinguished Professor and Associate Chair for Research and Healthcare Informatics. Dr. Brown is the former chief executive officer at START Treatment and Recovery Center. Mr. Bossert is a former opioid treatment program administrator and surveyor for the Commission on Accreditation of Rehabilitation Facilities. Dr. Dharia is a physician and the Director of Liver Services. Mr. Mukhopadhyay is a research assistant and doctoral student in Computational and Data-enabled Sciences and Engineering at University at Buffalo. |
| 4. Gender | Was the researcher male or female? | NA | Male: Talal, Dharia, Brown, Bossert, Mukhopadhyay  Female: Markatou, Grubbs, Ntiri-Reid, Houtsmuller |
| 5. Experience and training | What experience or training did the researcher have? | Manuscript page 5-6 | Dr. Talal is a physician-scientist with more than 20 years of experience in treating HCV. He serves on the NYS HCV Elimination Task Force and its HCV Guidelines Committee. He has published several qualitative studies. Ms. Ntiri-Reid leads integration of HIV, viral hepatitis, and harm reduction services. Zakiya Grubbs supports prevention and surveillance capacity building through NASTAD’s Online Technical Assistance Center for Viral Hepatitis. Dr. Houtsmuller was an Associate Director at PCORI and the trial’s program officer. Dr. Markatou was the trial’s lead biostatistician. Dr. Brown is the former chief executive officer at START, the oldest and largest minority-run substance use treatment program in the United States. Mr. Bossert was a former opioid treatment program administrator and accreditations surveyor. Dr. Dharia is a physician with several publications of qualitative studies. Mr. Mukhopadhyay is a research assistant and doctoral student in Computational and Data-enabled Sciences and Engineering. |
| *Relationship with participants* | | | |
| 6. Relationship established | Was a relationship established prior to study commencement? | Manuscript page 5 | Yes, all speakers were invited to participate in the workshop based on their roles and high degree of engagement in the randomized controlled trial of facilitated telemedicine. |
| 7. Participant knowledge of the interviewer | What did the participants know about the researcher? e.g. personal goals, reasons for doing the research | NA | NA |
| 8. Interviewer characteristics | What characteristics were reported about the interviewer/facilitator? e.g. bias, assumptions, reasons and interests in the research topic | Manuscript page 5-6 | Characteristics are described in Domain 1, *Personal Characteristics, questions 1-5.* |
| **Domain 2: Study design** | | | |
| *Theoretical framework* | | | |
| 9. Methodological orientation and Theory | What methodological orientation was stated to underpin the study? e.g. grounded theory,  discourse analysis, ethnography, phenomenology, content analysis | Manuscript page 9 | “Using a constructivist paradigm, we performed a thematic analysis to identify facilitated telemedicine considerations that overcame implementation challenges and delivered patient-centered care. Two investigators, Drs. Dharia and Talal, followed the 6-step framework for thematic analysis described by Braun and Clarke. Drs. Dharia and Talal have published several qualitative studies in Q1 journals and are well versed in the approaches to minimize bias in qualitative research. They became familiar with the data by independently reviewing the transcripts for accuracy and formatting. They independently and inductively coded the transcript data based on its relevance to facilitated telemedicine considerations that overcame implementation challenges and delivered patient-centered care. Subsequently, they independently grouped the initial codes into general categories. In a recursive process, they discussed code generation to identify and interpret repeated patterns of meaning and nuances derived from each presentation. They met frequently to discuss these patterns of meaning until a consensus was reached and lower-level themes were developed with supporting quotations. In the case of disagreements in the textual interpretations, they reviewed the transcripts until a consensus was reached.” |
| *Participant selection* | | | |
| 10. Sampling | How were participants selected? e.g. purposive, convenience, consecutive, snowball | Manuscript page 5 | All speakers were invited to participate in the workshop based on their roles and high degree of engagement in the trial. |
| 11. Method of approach | How were participants approached? e.g. face-to-face, telephone, mail, email | Manuscript page 6 | Speakers were invited by email. |
| 12. Sample size | How many participants were in the study? | Manuscript page 5 | The workshop was hosted by 2 planners, 1 moderator, and 7 speakers. |
| 13. Non-participation | How many people refused to participate or dropped out? Reasons? | Manuscript page 6 | “Two individuals, representing an expert in substance use disorder and a patient-participant with lived experience, were unable to participate.” |
| *Setting* | | | |
| 14. Setting of data collection | Where was the data collected? e.g. home, clinic, workplace | Manuscript page 8-9 | “The 3.5-hour workshop was recorded and transcribed by Zoom. All workshop speakers disseminated their successful approaches that overcame facilitated telemedicine implementation challenges. After the final set of transcripts was obtained, they were reviewed for completeness independently by two investigators with inconsistencies verified by listening to the recordings. While the transcripts did have identifiable quotations, those that were used in the analysis were deidentified. The workshop transcripts served as the raw data and the primary source for the thematic analysis.” |
| 15. Presence of non-participants | Was anyone else present besides the participants and researchers? | Manuscript page 4 | The audience participated in the workshop through 2 Q&A sessions. |
| 16. Description of sample | What are the important characteristics of the sample? e.g. demographic data, date. | Manuscript page 5 | All speakers were invited to participate in the workshop based on their roles and high degree of engagement in the trial. |
| *Data collection* | | | |
| 17. Interview guide | Were questions, prompts, guides provided by the authors? Was it pilot tested? | Manuscript page 8 | “Once the speakers had agreed to present at the workshop, they worked iteratively with the planning committee to obtain feedback on the presentations. When the presentations were finalized, each speaker uploaded the presentation to the AATOD website. Workshop speakers met in person prior to the workshop to review the presentations’ content and to promote cohesiveness and flow. Speakers reviewed logistical considerations immediately prior to the workshop, including timing, recording and moderation by NASTAD staff. Two question and answer sessions were held during the workshop that were moderated by Ms. Grubbs. During the introductory remarks, the moderator discussed the objectives of the conference, the context of HCV with a special emphasis on the importance of HCV elimination, and specific areas of discussion for the audience. To minimize recollection bias, the question-and-answer sessions occurred immediately after the workshop presentations. As the workshop presenters had extensive experience working collaboratively to achieve the trial’s goals, respondent bias was minimized. On the other hand, however, the moderator was not previously known to the workshop presenters and represented a public health advocacy perspective, which encouraged discussion of a diverse array of perspectives. To ensure question comprehension, the moderator repeated the question prior to eliciting a response. The moderator also sought responses from multiple workshop presenters as appropriate. After completion of the workshop, the planning committee met to discuss topics for manuscript development. The primary focus of the planning committee was the thematic analysis of the workshop presentation transcripts.” |
| 18. Repeat interviews | Were repeat interviews carried out? If yes, how many? | NA | NA |
| 19. Audio/visual recording | Did the research use audio or visual recording to collect the data? | Manuscript page 8 | “The 3.5-hour workshop was recorded and transcribed by Zoom.” |
| 20. Field notes | Were field notes made during and/or after the interview or focus group? | NA | Yes, some speakers took field notes during the workshop. |
| 21. Duration | What was the duration of the interviews or focus group? | Manuscript page 8 | “The 3.5-hour workshop was recorded and transcribed by Zoom.” |
| 22. Data saturation | Was data saturation discussed? | Manuscript page 6 | “We used investigator and data source triangulation to understand when we had achieved sample saturation.” |
| 23. Transcripts returned | Were transcripts returned to participants for comment and/or correction? | Manuscript page 9 | “Once a draft of concepts had been derived and agreed upon, the lower-level themes were discussed and reviewed with all co-authors in a series of meetings. Using a consensus and iterative approach, lower-level themes were defined and evolved into higher-level themes. Verbatim quotes from the workshop presentations were highlighted to support the themes. All co-authors reviewed the final themes and supporting quotes for agreement. All co-authors were active participants in the data-driven thematic analysis.” |
| **Domain 3: analysis and findings** | | | |
| *Data analysis* | | | |
| 24. Number of data coders | How many data coders coded the data? | Manuscript page 9 | “Two investigators, Drs. Dharia and Talal, followed the 6-step framework for thematic analysis described by Braun and Clarke [18,19]. Drs. Dharia and Talal have published several qualitative studies in Q1 journals [3,9,20-25] and are well versed in the approaches to minimize bias in qualitative research. They became familiar with the data by independently reviewing the transcripts for accuracy and formatting. They independently and inductively coded the transcript data based on its relevance to facilitated telemedicine considerations that overcame implementation challenges and delivered patient-centered care. Subsequently, they independently grouped the initial codes into general categories. In a recursive process, they discussed code generation to identify and interpret repeated patterns of meaning and nuances derived from each presentation. They met frequently to discuss these patterns of meaning until a consensus was reached and lower-level themes were developed with supporting quotations.” |
| 25. Description of the coding tree | Did authors provide a description of the coding tree? | Manuscript page 9 | “Two investigators, Drs. Dharia and Talal, followed the 6-step framework for thematic analysis described by Braun and Clarke [18,19]. Drs. Dharia and Talal have published several qualitative studies in Q1 journals [3,9,20-25] and are well versed in the approaches to minimize bias in qualitative research. They became familiar with the data by independently reviewing the transcripts for accuracy and formatting. They independently and inductively coded the transcript data based on its relevance to facilitated telemedicine considerations that overcame implementation challenges and delivered patient-centered care. Subsequently, they independently grouped the initial codes into general categories. In a recursive process, they discussed code generation to identify and interpret repeated patterns of meaning and nuances derived from each presentation. They met frequently to discuss these patterns of meaning until a consensus was reached and lower-level themes were developed with supporting quotations.” |
| 26. Derivation of themes | Were themes identified in advance or derived from the data? | Manuscript page 9 | Themes were derived from the data. |
| 27. Software | What software, if applicable, was used to manage the data? | NA | NA |
| 28. Participant checking | Did participants provide feedback on the findings? | Manuscript page 9 | “Once a draft of concepts had been derived and agreed upon, the lower-level themes were discussed and reviewed with all co-authors in a series of meetings. Using a consensus and iterative approach, lower-level themes were defined and evolved into higher-level themes. Verbatim quotes from the workshop presentations were highlighted to support the themes. All co-authors reviewed the final themes and supporting quotes for agreement. All co-authors were active participants in the data-driven thematic analysis. We acknowledge that the experiences and interactions of the co-authors may have influenced the thematic analysis in this constructivist paradigm.” |
| *Reporting* | | | |
| 29. Quotations presented | Were participant quotations presented to illustrate the themes / findings? Was each quotation identified? e.g. participant number | Manuscript page 9-17 | Yes, quotations are presented throughout the Results section and Table 1. |
| 30. Data and findings consistent | Was there consistency between the data presented and the findings? | Manuscript page 9-17 | Yes, there was consistency between the data presented and the findings. Additionally, novel concepts or themes were developed from the thematic analysis of the workshop presentation transcripts. |
| 31. Clarity of major themes | Were major themes clearly presented in the findings? | Manuscript page 9 | “We developed three themes from the textual analysis of the workshop presentations.” |
| 32. Clarity of minor themes | Is there a description of diverse cases or discussion of minor themes? | NA | NA |

### Supplemental references

1. Talal AH, Markatou M, Sofikitou EM, Brown LS, Perumalswami P, Dinani A, et al. Patient-centered HCV care via telemedicine for individuals on medication for opioid use disorder: Telemedicine for Evaluation, Adherence and Medication for Hepatitis C (TEAM-C). Contemp Clin Trials 2022;112:106632. [10.1016/j.cct.2021.106632][PMID:34813962]

2. Talal AH, Jaanimägi U, Davis K, Bailey J, Bauer BM, Dharia A, et al. Facilitating engagement of persons with opioid use disorder in treatment for hepatitis C virus infection via telemedicine: stories of onsite case managers. J Subst Abuse 2021:108421. [DOI:10.1016/j.jsat.2021.108421][PMID:34134875]

3. Talal AH, Markatou M, Liu A, Perumalswami PV, Dinani AM, Tobin JN, et al. Integrated Hepatitis C-Opioid Use Disorder Care Through Facilitated Telemedicine: A Randomized Trial. JAMA 2024;331(16):1369-1378. [10.1001/jama.2024.2452][PMC10993166]

4. Talal AH, Sofikitou EM, Wang K, Dickerson S, Jaanimagi U, Markatou M. High satisfaction with patient-centered telemedicine for hepatitis C virus delivered to substance users: a mixed-methods study. Telemed J E Health 2023;29(3):395-407. [DOI:10.1089/tmj.2022.0189][PMID:35925809]
