# Supplementary material for: Facilitated Telemedicine as a Patient-Centered, Sociotechnical Intervention to Integrate Hepatitis C Treatment Into Opioid Treatment Programs and Overcome the Digital Divide Among Underserved Populations: Qualitative Study
Source: JMIR Public Health Surveill. 2025 Jul 16;11:e68854. doi: 10.2196/68854 (PMC12286564; doi:10.2196/68854)
Supplement: Multimedia Appendix 2 [file publichealth-v11-e68854-s002.docx]

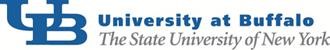


**University at Buffalo Institutional Review Board (UBIRB)**

Office of Research Compliance | Clinical and Translational Research Center Room 5018

875 Ellicott St. | Buffalo, NY 14203

UB Federalwide Assurance ID#: FWA00008824

# Complete Research Protocol (HRP-503)

## Table of Contents

Template Instructions.......................................................................................................... 2

1.0 Objectives ................................................................................................................ 4

2.0 Scientific Endpoints ................................................................................................. 5

3.0 Background .............................................................................................................. 6

4.0 Study Design ............................................................................................................ 9

5.0 Local Number of Subjects ..................................................................................... 10

6.0 Inclusion and Exclusion Criteria ............................................................................ 11

7.0 Vulnerable Populations .......................................................................................... 13

8.0 Eligibility Screening .............................................................................................. 14

9.0 Recruitment Methods ............................................................................................. 15

10.0 Procedures Involved............................................................................................... 17

11.0 Study Timelines ..................................................................................................... 24

12.0 Setting .................................................................................................................... 25

13.0 Community-Based Participatory Research ............................................................ 26

14.0 Resources and Qualifications ................................................................................. 27

15.0 Other Approvals ..................................................................................................... 29

16.0 Provisions to Protect the Privacy Interests of Subjects .......................................... 29

17.0 Data Management and Analysis ............................................................................ 30

18.0 Confidentiality ....................................................................................................... 33

1. Confidentiality of Study Data ............................................................................ 33
2. Confidentiality of Study Specimens ................................................................... 36

19.0 Provisions to Monitor the Data to Ensure the Safety of Subjects .......................... 38

20.0 Withdrawal of Subjects .......................................................................................... 39

21.0 Risks to Subjects ................................................................................................ 40

22.0 Potential Benefits to Subjects ................................................................................ 42

23.0 Compensation for Research-Related Injury ........................................................... 42

24.0 Economic Burden to Subjects ................................................................................ 42

25.0 Compensation for Participation ............................................................................. 43

26.0 Consent Process ..................................................................................................... 44 27.0 Waiver or Alteration of Consent Process ............................................................... 48 28.0 Process to Document Consent ............................................................................... 50 29.0 Multi-Site Research (Multisite/Multicenter Only).............................................. . 51.... 30.0 Banking Data or Specimens for Future Use ....................................................52 31.0 Drugs or Devices........................................................................................................54

32.0 Humanitarian Use Devices .................................................................................... 55

Page **1** of **58**

***Template Instructions***

***Sections that do not apply:***

- *In several sections, the addition of checkboxes for* ***Not Applicable*** *have been added to the template as responses.* o *If an N/A checkbox is present, select the appropriate justification from the list.*
  - *If an N/A checkbox is not present, or if none of the existing checkboxes apply to your study, you must write in your own justification.*
- *In addition:*
  - *For research where the only study procedures are records/chart review: Sections 19, 20, 22, 23, 24, 25, 31, and 32 do not apply.* o *For exempt research: Sections 31 and 32 do not apply.*

***Studies with multiple participant groups:***

- *If this study involves multiple participant groups (e.g. parents and children), provide information in applicable sections for each participant group. Clearly label responses when they differ. For example:*

Response:

Intervention Group: management of hepatitis C virus (HCV) infection via telemedicine

Control Group:

Usual care treatment of HCV.

***Formatting:***

- *Do not remove template instructions or section headings when they do not apply to your study.*

*If you are pasting information from other documents using the “Merge Formatting” Paste option will maintain the formatting of the response boxes.*

***Amendments:***

- *When making modifications or revisions to this and other documents, use the* ***Track Changes*** *function in Microsoft Word.*
- *Update the version date or number* ***on Page 3.***

**PROTOCOL TITLE:**

*Include the full protocol title.*

Response:

*Stepped-Wedge Randomized Control Trial to Compare Integrated, Co-located,*

*Telemedicine-based Treatment Approach for Hepatitis C Virus (HCV)*

*Management for Individuals on Opiate Agonist Treatment and Usual Care*

*Treatment of HCV of individuals on Opiate Agonist Treatment*

**PRINCIPAL INVESTIGATOR:**

*Name*

*Department*

*Telephone Number*

*Email Address*

| Response: Andrew Talal MD.  University at Buffalo Department of Medicine  Clinical and Translational Research Center  716 888-4737 ahtalal@buffalo.edu |
| --- |

**VERSION:**

*Include the version date or number.*

Response: March 19, 2025

Version 24

**GRANT APPLICABILITY:**

*Indicate whether this protocol is funded by a grant (e.g. NIH, foundation grant). For a grant with multiple aims, indicate which aims are covered by this research proposal.*

*NOTE: This question does not apply to studies funded by a sponsor contract.*


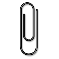
 *Include a copy of the grant proposal with your submission.*

Response: This protocol is funded by an award from the Patient Centered Outcomes Research Institute (PCORI).

|  |
| --- |

**RESEARCH REPOSITORY:**

*Indicate where the research files will be kept, including when the study has been closed. The repository should include, at minimum, copies of IRB correspondence (approval, determination letters) as well as signed consent documents. This documentation should be maintained for 3 years after the study has been closed.*

Response: All study documentation will be kept at the Clinical and Translational Research Center for a minimum of 3 years after the study has been completed.

*Location: University at Buffalo Dept. of Medicine*

*Address: 875 Ellicott St. Buffalo, N.Y. 14203 Rm 6035*

*Department: Dept. of Medicine*

## 1.0 Objectives

*1.1 Describe the purpose, specific aims, or objectives of this research.*

Response: This study aims to implement information technology (telemedicine) for improvement in health care delivery to the difficult to reach and underserved patient population of people with substance use disorders (PWSUD) who are on opiate agonist treatment (OAT).

Primary specific aim: To compare the effectiveness of a patient-centered, OAT-integrated telemedicine-based approach for management and delivery of HCV treatment to PWSUD versus usual care, referral to an offsite location for HCV management. The effectiveness will be expressed through the primary patient centered and clinical outcome, achievement of viral eradication or sustained virologic response (SVR), defined as undetectable HCV RNA 12 weeks post treatment cessation.

Our secondary aims are:

a. Comparison of treatment initiation rates between the two arms, as measured by the proportion of patients that take an initial medication dose. b. Comparison of treatment completion rates between the two arms.

1. Comparison of patient satisfaction with health care delivery between the two arms as assessed through the Hepatitis C Patient Satisfaction Questionnaire, Mini Mental Health Screen, National Institute on Drug Abuse Quick Screen, Drug Abuse Screening Test and Socio-demographics Survey.
2. Comparison of treatment adherence rates between the two arms, as measured by attendance at scheduled specialists, telemedicine visits, and medication adherence.

| e. Resource utilization between the two arms | | | | | | | | | | |
| --- | --- | --- | --- | --- | --- | --- | --- | --- | --- | --- |
| \| f. Association between social determinants of health and pursuit of HCV \| \| --- \| | | | | | | | | |  | |
| \| treatment in both arms. \| \| --- \| | | |  | | | | | |  |  |
|  | | |  |  |  |  |  |  |  |  |
| Interview substudy: | |  |  |  |  |  |  |  |  |  |
| \| g) Understand from the patient point-of-view the barriers and facilitators \| \| --- \| | | | | | | | |  | |  |
| \| for pursing telemedicine treatment of HCV. \| \| --- \| | | | |  | | | |  |  |  |
|  | | | |  |  |  |  |  |  |  |
| \| h) Understand the role that knowledge of new treatments or innovative \| \| --- \| | | | | | | |  |  |  |  |
| technologies play in patient’s willingness to be treated for HCV. | | | | |  | |  |  |  |  |
| \| i) From the staff point of view, what are the barriers and facilitators of \| \| --- \| | | | | | |  | | | | |
| \| establishing telemedicine as a method to integrate behavioral and medical \| \| --- \| | | | | | | | | | |  |
| \| treatment. \| \| --- \| |  | | | | | | | | |  |
|  |  |  |  |  |  |  |  |  |  |  |

*1.2 State the hypotheses to be tested, if applicable.*

*NOTE: A hypothesis is a specific, testable prediction about what you expect to happen in your study that corresponds with your above listed objectives.*

Response:

The primary hypothesis is a patient-centered OAT-integrated, telemedicine-based approach for delivery of HCV care that addresses PWSUDs’ specific needs will lead to improved treatment outcomes compared to usual care, which in most cases we assume will be referral to an offsite location for HCV management.

## 2.0 Scientific Endpoints

*2.1 Describe the scientific endpoint(s), the main result or occurrence under study.*

*NOTE: Scientific endpoints are outcomes defined before the study begins to determine whether the objectives of the study have been met and to draw conclusions from the data. Include primary and secondary endpoints. Some example endpoints are: reduction of symptoms, improvement in quality of life, or survival. Your response should* ***not*** *be a date.*

Response:

| o | Achievement of viral eradication defined as undetectable HCV RNA for  12 weeks post treatment cessation for those patients treated via telemedicine |
| --- | --- |
| o | Improvement in quality of life |
| o | Restore hepatic function |
| o | Decrease hepatic failure and incidence of transplants |
| o | Diminished pool of HCV-infected people and consequently future HCV incidence and prevalence. |

## 3.0 Background

*3.1 Provide the scientific or scholarly background, rationale, and significance of the research based on the existing literature and how it will contribute to existing knowledge. Describe any gaps in current knowledge. Include relevant preliminary findings or prior research by the investigator.*

Response:

HCV infection is a leading cause of chronic liver disease affecting more than 120 million people globally^1^ and ~5 million in the United States (US)^2^. Approximately 80% of HCV-exposed individuals develop chronic infection that can progress to cirrhosis, liver cancer, and death. As 50% to 75% of those infected are unaware of their status^3^, the impetus to diagnose and to treat chronic HCV is tremendous. Currently, HCV is the leading indication for liver transplantation in the US^4^. The prevalence of HCV-related liver cirrhosis is predicted to increase from 25% in 2010 to 45% by 2030 and liver-related deaths are projected to increase by 175% over the next decade^5^. Injection drug use is the primary route of HCV transmission; estimated HCV prevalence among PWSUD is 30% to 70% while incidence ranges from 16% to 42% per year^6^,^7^. Of those with chronic HCV infection, 56% have a history of injection drug use^8^.

Recent therapeutic advances have radically improved HCV treatment.

Using a combination of orally administered, direct acting antivirals (DAAs), treatment is curative in more than 90% of individuals with virtually no side effects and substantially shortened treatment duration (i.e., 8 to 12 weeks)^9^,^10^,^11^. Previously, interferon (IFN) was prescribed for HCV and had significant, frequently treatment-limiting side effects^12^,^13^.

Despite these advances, DAA dissemination to PWSUD has been limited. Consequently, new patient-centered approaches are critical to deliver these medications to those most desperate for treatment. PWSUD referral to an HCV specialist is the current usual HCV management strategy. Unfortunately, despite the majority (>70%) of PWSUD indicating willingness to pursue HCV treatment, only 21% to 65% of HCV seropositive PWSUD have been evaluated for HCV and only a minor percentage (1% to 6%) actually receives therapy^14^,^15^,^9^,^10^. The importance of HCV treatment among PWSUD will only increase as a result of an increase in acute HCV cases among injection drug users < 30 years^11^.

Treatment of substance use: Of treatments available for opiate dependence, methadone maintenance when combined with medical, psychiatric, and socioeconomic support services has the highest probability of effectiveness^16^. Because of a concern of illegal sale and distribution of methadone, most patients receiving methadone through an OAT program are required to attend clinic appointments 3 to 6 days per week^16^. The frequency of required OAT program visits provides an opportunity for otherwise hard-to-reach patients to access health care services, an important consideration as many PWSUDs experience multiple health problems, including higher rates of viral hepatitis, HIV and sexually transmitted infections compared to the general population^17^. Many OAT programs also represent a primary health care site for their patients.

For this study we are implementing and evaluating a patient centered, OAT program-integrated telemedicine approach for the management and delivery of HCV care to diverse PWSUD populations compared with usual care (referral). The patient-centered telemedicine approach, based on diffusion of innovations theory (DOI), is key for the intervention and treatment of HCV, and ultimately other frequent co-morbid conditions that affect PWSUD^18^.

Interview substudy:

Patients: As part of our ongoing PCORI study, we have collected patient surveys that assess their responses and reactions to telemedicine or referral -based care for HCV. We have also recently completed a study that identified themes derived from case managers’ stories of HCV treatment. We identified three themes as being important for pursuit of HCV care: 1) trust in the opioid treatment program (OTP), 2) identification of competing priorities, and 3) personalizing care so that identified priorities are addressed. Satisfactorily addressing these issues enables pursuit of HCV care (Talal, Jaanimägi, et al., 2020; Zeremski et al., 2014).

We now propose to pursue a study of patient interviews and focus groups for a more in depth assessment of their reactions to telemedicine and referral-based care. Our experiences have shown that direct patient contact is the most effective method to assess reactions to an intervention in this population (Talal et al., 2019). We will utilize the interviews to pursue a mixed qualitative/quantitative manuscript combining the patient reaction data with the already collected survey data.

Staff – In 2013, we published a paper on clinical staff attitudes toward onsite HCV treatment within the OTP (Talal et al., 2013). In other work, we have shown that knowledge of HCV natural history and treatment improves the likelihood of primary care providers pursuing HCV screening (Samuel, Martinez, Chen, Markatou, & Talal, 2018).Our goal now is to update our knowledge of barriers and facilitators to HCV treatment pursuit among staff at substance use treatment programs, to identify barriers to telemedicine and how they have been overcome, and considerations and lessons learned about telemedicine implementation from the point of view of the staff working at or leading OTPs (Talal, Sofikitou, et al., 2020).

*3.2 Include complete citations or references.*

Response:

1. World Health Organization. *Hepatitis C. Fact Sheet No. 164*.

[http://www.who.int/mediacentre/factsheets/fs164/en/.](http://www.who.int/mediacentre/factsheets/fs164/en/) Accessed August 21, 2013.

1. Chak E, Talal AH, Sherman KE, Schiff ER, Saab S. Hepatitis C virus infection in USA: an estimate of true prevalence. *Liver Int.* 2011;31(8):1090-1101.
2. Smith BD, Morgan RL, Beckett GA, et al. Recommendations for the identification of chronic hepatitis C virus infection among persons born during 1945-1965. *MMWR Recomm Rep.* 2012;61(RR-4):1-32.
3. Brown RS. Hepatitis C and liver transplantation. *Nature.* 2005;436(7053):973978.
4. Davis GL, Alter MJ, El-Serag H, Poynard T, Jennings LW. Aging of hepatitis C virus (HCV)-infected persons in the United States: a multiple cohort model of HCV prevalence and disease progression. *Gastroenterology.* 2010;138(2):513521, 521 e511-516.
5. Amon JJ, Garfein RS, Ahdieh-Grant L, et al. Prevalence of hepatitis C virus infection among injection drug users in the United States, 1994-2004. *Clin Infect Dis.* 2008;46(12):1852-1858.
6. Edlin BR, Carden MR. Injection drug users: the overlooked core of the hepatitis C epidemic. *Clin Infect Dis.* 2006;42(5):673-676.
7. Armstrong GL, Wasley A, Simard EP, McQuillan GM, Kuhnert WL, Alter MJ. The prevalence of hepatitis C virus infection in the United States, 1999 through 2002. *Ann Intern Med.* 2006;144(10):705-714.
8. Grebely J, Raffa JD, Lai C, et al. Low uptake of treatment for hepatitis C virus infection in a large community-based study of inner city residents. *J Viral Hepat.* 2009;16(5):352-358.
9. Schackman BR, Teixeira PA, Beeder AB. Offers of hepatitis C care do not lead to treatment. *J Urban Health.* 2007;84(3):455-458.
10. Zibbell JE, Iqbal K, Patel RC, et al. Increases in hepatitis C virus infection related to injection drug use among persons aged </=30 years - Kentucky, Tennessee, Virginia, and West Virginia, 2006-2012. *MMWR Morb Mortal Wkly Rep.* 2015;64(17):453-458.
11. Fried TR, Bradley EH, Towle VR, Allore H. Understanding the treatment preferences of seriously ill patients. *N Engl J Med.* 2002;346(14):1061-1066.
12. Manns MP, McHutchison JG, Gordon SC, et al. Peginterferon alfa-2b plus ribavirin compared with interferon alfa-2b plus ribavirin for initial treatment of chronic hepatitis C: a randomised trial. *Lancet.* 2001;358(9286):958-965.
13. Mehta SH, Genberg BL, Astemborski J, et al. Limited uptake of hepatitis C treatment among injection drug users. *J Community Health.* 2008;33(3):126-133.
14. Grebely J, Genoway KA, Raffa JD, et al. Barriers associated with the treatment of hepatitis C virus infection among illicit drug users. *Drug Alcohol Depend.* 2008;93(1-2):141-147.
15. Vocal-NY. *Beyond Methadone: Improving Health and Empowering Patients in Opioid Treatment Programs.* [www.methadonemaintenance.net,](http://www.methadonemaintenance.net/) 2011.
16. Masson CL, Sorensen JL, Batki SL, Okin R, Delucchi KL, Perlman DC. Medical service use and financial charges among opioid users at a public hospital. *Drug Alcohol Depend.* 2002;66(1):45-50.
17. Rogers EM. *Diffusion of innovations.* 5th ed. New York: Free Press; 2003.

| 19. | Samuel, S. T., Martinez, A. D., Chen, Y., Markatou, M., & Talal, A. H. (2018). Hepatitis C virus knowledge improves hepatitis C virus screening practices among primary care physicians. World Journal of Hepatology, 10(2), 319. |  |  |
| --- | --- | --- | --- |
| 20. | Talal, A. H., Dimova, R. B., Seewald, R., Peterson, R. H., Zeremski, M., Perlman, D. C., & Des Jarlais, D. C. (2013). Assessment of methadone clinic staff attitudes toward hepatitis C evaluation and treatment. Journal of Substance Abuse Treatment, 44(1), 115-119. |  |  |
| 21. | Talal, A. H., Jaanimägi, U., Davis, K., Bailey, J., Bauer, B. M., Dharia, A.,  . . . Dickerson, S. S. (2020). Factors Affecting Persons with Opioid Use  Disorder Engagement in Treatment for Hepatitis C Virus Infection via Telemedicine. Journal of Substance Abuse Treatment, Manuscript in Preparation. |  |  |
| 22. | Talal, A. H., McLeod, A., Andrews, P., Nieves-McGrath, H., Chen, Y., Reynolds, A., . . . Brown, L. S. (2019). Patient reaction to telemedicine for clinical management of hepatitis C virus integrated into an opioid treatment program. Telemedicine and e-Health, 25(9), 791-801. |  |  |
| 23. | Talal, A. H., Sofikitou, E. M., Jaanimägi, U., Zeremski, M., Tobin, J. N.,  & Markatou, M. (2020). An Implementation Framework for PatientCentered Telemedicine for Vulnerable Populations. Journal of Biomedical Informatics, Invited Revision. |  |  |
| 24. | Zeremski, M., Dimova, R. B., Zavala, R., Kritz, S., Lin, M., Smith, B. D., . . . Talal, A. H. (2014). Hepatitis C virus-related knowledge and willingness to receive treatment among patients on methadone maintenance. J Addict Med, 8(4), 249-257. doi:10.1097/ADM.0000000000000041 | | |
|  | |  |  |

## 4.0 Study Design

*4.1 Describe and explain the study design (e.g. case-control, cross-sectional, ethnographic, experimental, interventional, longitudinal, observational).*

Response: Our study will be conducted as a non-blinded Stepped Wedge Cluster Randomized Controlled Trial with two arms: Onsite HCV management through telemedicine versus HCV management through usual care, which in most cases will be referral to an offsite liver specialist (Referral). The arm assignment will be at the cluster (clinic) level. After an initial period 9 months) in which all OAT clinics implement the control intervention (usual care), at regular intervals (i.e., the “steps”) of 9 months duration each, one group of OAT clinics will be randomized to cross over from the Usual Care arm to the Telemedicine arm. In this way, there will be enough time for implementation and assessment of the intervention within each time period. The process continues until all OAT clinics have crossed over to implement telemedicine, and thus the clinics contribute data to both interventions. Board-eligible or certified providers will manage HCV treatment. All sites are allocated to a single cluster. Therefore, no participant cross-over between the arms is permitted.

Interview substudy:

This is an observational study consisting of interviews and focus groups.

## 5.0 Local Number of Subjects

*5.1 Indicate the total number of subjects that will be enrolled or records that will be reviewed locally.*

Response: The University at Buffalo Dept. of Medicine is the Sponsor of the study initiating the study design and managing its implementation as well as data analysis. Therefore, UB staff will not be directly consenting or enrolling subjects. Screening, consenting and enrollment of subjects will be at each participating

OAT clinic. Our target sample size is 624 OAT program-maintained patients from 12 clinics throughout the state of NY Interview substudy:

Patients-We will plan to interview up to 25 patients who participated in the parent study (approximately 2 per clinic).

Staff: We will plan to interview/conduct focus groups with up to 200 individuals across the study clinics.

We may also contact sites that declined to participate in the parent study to assess the reasons for non-participation. These individuals would likely represent the leadership at these sites, including administrators, providers, lawyers or clinic directors.

We are also trying to interview patients who were invited to participate in the study but declined.

*5.2 If applicable, indicate how many subjects you expect to screen to reach your target sample (i.e. your screen failure rate).*

Response: We estimate that approximately 2534 patients (~130 patients/clinic) will be screened.

*5.3 Justify the feasibility of recruiting the proposed number of eligible subjects within the anticipated recruitment period. For example, how many potential subjects do you have access to? What percentage of those potential subjects do you need to recruit?*

Response:

Currently, there are 126 OAT programs in New York State. Estimated HCV (antibody) prevalence among PWID ranges from 30% to 70%, depending on frequency and duration of use, while incidence ranges from 16% to 42% per year.

Interview Substudy

Most of the potential participants are subjects from the parent study, so many of them are either still in regular contact with the study staff or have contact information available.

For staff, we will work closely with the clinic leadership at each site to tailor staff recruitment to be consistent with local procedures. We have been working with all sites since late 2016, and the parent study is very well known to clinic leadership and staff. Many staff have been involved in either HCV medication dispensing, supporting patients through their treatment, or identifying patients for recruitment.

We will also seek to conduct interviews or focus groups with staff at sites that might have declined study participation. Additional interviews or focus groups might be conducted with state agencies that oversee telemedicine or substance use treatment, such as New York State Dept of Health and the Office of Addiction Services and Supports (OASAS). The goals of these discussions will be to understand facilitators and barriers to telemedicine implementation and how these might have changed as a result of the COVID-19 pandemic.

## 6.0 Inclusion and Exclusion Criteria

*6.1 Describe the criteria that define who will be* ***included*** *in your final study sample.*

*NOTE: This may be done in bullet point fashion.*

Response: The inclusion criteria:

1. HCV antibody detected
2. Ability and willingness of subject or legal representative to provide written informed consent.
3. 18 years of age or older
4. A minimum of 6-month enrollment in the OAT program
5. Likely to be adherent to the therapeutic regimen
6. Treatment must be covered by medical insurance

Interview Substudy:

Subjects from the parent study or staff that worked at the study site clinics during the study period will be eligible. We also may approach current or former members of our.patient advisory committee that is operational at each site and ask them to participate in focus groups or interviews.

Additional interviews may be conducted with staff from other substance use treatment facilities or governmental agencies that participate in telemedicine or substance use treatment

Patients at the clinic who were invited to participate in the study but declined are eligible to be interviewed in a single session, but will not participate in any other aspect of the study.

*6.2 Describe the criteria that define who will be* ***excluded*** *from your final study sample.*

*NOTE: This may be done in bullet point fashion.*

Response:

1. Mental instability or incompetence, such that the validity of the informed consent or ability to be compliant with the study is uncertain.
2. <18 years of age
3. < 6 months enrolled in OAT program.
4. Non-compliance with methadone therapeutic regimen at the Opiate Agonist Treatment program, defined as three consecutive missed appointments that will result in a discussion between the principal investigator at the site and the study participant to determine the reason for the missed appointments. Continuation in the study for the participant will be assessed on a case-bycase basis between the study PI and the site sub-PI.
5. Lack of medical insurance coverage
6. Ineligibility for HCV treatment
7. Active treatment for HCV at the time of the study enrollment
8. HIV positive- not on stable antiviral therapy
9. Enrolled subjects will be withdrawn if they are discharged from their clinic or their HCV medication administration lapses for 1 month or more
   1. *Indicate specifically whether you will include any of the following special populations in your study using the checkboxes below.*

***NOTE: Members of special populations may not be targeted for enrollment in your study unless you indicate this in your inclusion criteria.***

Response: we will not enroll any special populations in our study

☐ Adults unable to consent

☐ Individuals who are not yet adults (infants, children, teenagers)

| ☐ | Pregnant women |
| --- | --- |
| ☒ | Prisoners |

- 1. *Indicate whether you will include non-English speaking individuals in your study.* ***Provide justification if you will exclude non-English speaking individuals.***

*In order to meet one of the primary ethical principles of equitable selection of subjects, non-English speaking individuals may* ***not*** *be routinely excluded from research as a matter of convenience.*

*In cases where the research is of therapeutic intent or is designed to investigate areas that would necessarily require certain populations who may not speak English, the researcher is required to make efforts to recruit and include non-English speaking individuals. However, there are studies in which it would be reasonable to limit subjects to those who speak English. Some examples include pilot studies, small unfunded studies with validated instruments not available in other languages, studies with numerous questionnaires, and some non-therapeutic studies which offer no direct benefit.*

Response:

Study materials will be offered in both English and Spanish, the two languages that are spoken by the vast majority of OAT program patients in NY State.

At least one of the patients who declined participation and will be invited to participate in a single interview is Spanish speaking.

**7.0** **Vulnerable Populations**

*If the research involves special populations that are considered vulnerable,* ***describe the safeguards included to protect their rights and welfare.***

*NOTE: You should refer to the appropriate checklists, referenced below, to ensure you have provided adequate detail regarding safeguards and protections. You do not, however, need to provide these checklists to the IRB.*

*7.1 For research that involves* ***pregnant women,*** *safeguards include: NOTE CHECKLIST: Pregnant Women (HRP-412)*

Response:

☒ **N/A**: This research does not involve pregnant women.

*7.2 For research that involves* ***neonates of uncertain viability or non-viable neonates,*** *safeguards include:*

*NOTE CHECKLISTS: Non-Viable Neonates (HRP-413), or Neonates of Uncertain Viability (HRP-414)*

Response:

| ☒ | **N/A:** This research does not involve non-viable neonates or neonates of uncertain viability. |
| --- | --- |

*7.3 For research that involves* ***prisoners****, safeguards include: NOTE CHECKLIST: Prisoners (HRP-415)*

*Response:* We do not plan to study prisoners specifically, but since many methadone-maintained patients cycle through the correctional system quite frequently, there will be instances when we have study subjects who become incarcerated. No study related procedures will occur during the time of the incarceration, but will resume if possible after the subject is released. No data will be collected on the subject during the time of incarceration. Subjects will not be recruited while incarcerated. If a subject is incarcerated while being treated for HCV, the treatment may be continued as standard of care during the incarceration. Subjects will only be withdrawn from the study for incarceration if it causes them to meet the overall criteria for withdrawal from the study: they are discharged from the clinic or if their HCV medication administration lapses for 1 month or more.

If incarcerated subjects are withdrawn from the study, the study data collected on them up to the point of withdrawal will still be used for the study.

☐ **N/A:** This research does not involve prisoners.

*6.5 7.4 For research that involves* ***persons who have not attained the legal age for consent to treatments or procedures involved in the research (“children”)****, safeguards include:*

*NOTE CHECKLIST: Children (HRP-416)*

Response:

☒ **N/A:** This research does not involve persons who have not attained the legal age for consent to treatments or procedures (“children”).

*6.6 7.5 For research that involves* ***cognitively impaired adults****, safeguards include: NOTE CHECKLIST: Cognitively Impaired Adults (HRP-417)*

Response:

☒ **N/A:** This research does not involve cognitively impaired adults.

*7.6 Consider if other specifically targeted populations such as students, employees of a specific firm, or educationally or economically disadvantaged persons are vulnerable.* ***Provide information regarding their safeguards and protections, including safeguards to eliminate coercion or undue influence.***

| Response: there will not be students, employees of a specific firm or educationally or economically disadvantaged persons targeted Interview substudy:  Subjects from our PCORI study and staff at the OTP clinics will be targeted, but the voluntary nature of participation will be stressed during the consent process. This will also be stressed to sites that may not be participating in or who declined participation in the PCORI study as well. |
| --- |

## 8.0 Eligibility Screening

*8.1 Describe* ***screening procedures*** *for determining subjects’ eligibility. Screening refers to determining if prospective participants meet inclusion and exclusion criteria.*


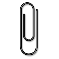
 *Include all relevant screening documents with your submission (e.g. screening protocol, script, questionnaire).*

Response: By New York State regulation, all OAT programs are required to screen for the presence of HCV antibody on an annual basis. Consequently, potential subjects in this study will be identified from the health record at each of the participating clinics for the presence of HCV antibody. In the case that a clinic does not screen for HCV, we will provide HCV antibody and HCV RNA screening as part of this protocol, and provide the necessary screening kits to the clinic.

☐ **N/A:**

## 9.0 Recruitment Methods

☐ **N/A:** This is a records review only, and subjects will not be recruited. NOTE: If you select this option, please make sure that all records review procedures and inclusion/exclusion screening are adequately described in other sections.

*9.1 Describe when, where, and how potential subjects will be recruited.*

*NOTE: Recruitment refers to how you are identifying potential participants and introducing them to the study. Include specific methods you will use (e.g.*

*searching charts for specific ICD code numbers, Research Participant Groups, posted advertisements, etc.).*

Response: By regulation, all OAT programs in NYS are required to screen for the presence of HCV antibody on an annual basis.

Consequently, potential subjects in this study will be identified from the health record at each of the participating clinics for the presence of HCV antibody. A partial HIPAA waiver will be obtained to allow for review of potential subjects HCV antibody status. We will offer screening for HCV antibody and HCV RNA for any patients or clinics that have not had access to these tests. A brochure and flyer will be distributed to all sites to enhance recruitment. A video simulating a telemedicine visit within a methadone clinic will be available for patients to view when deciding on participation within the telemedicine arm. The goal is to enhance understanding how telemedicine can be incorporated into the daily flow of the subject’s routine when coming for dosing of methadone. The video produced by the University at Buffalo Center for the Arts will be completed using actors and clinic setting not involved in the study.

Recruitment of subjects to participate in the dissemination of results (interviews and public speaking opportunities) will be done either with a phone call or at the subject’s study visit. Interview substudy:

Patients

We will plan to interview up to 25 patients who participated in the parent study (approximately 2 per clinic) utilizing purposive sampling. We will approach PCORI parent study participants who have had particularly interesting or meaningful experiences as a result of the treatment modality of telemedicine. Potential subjects will be contacted by the case manager who intereacted with them in the parent study, either in person or by text or phone call or email, depending on the known preferences of the individual.

Staff

We will plan to interview up to 200 staff who participated in or were involved with the parent study utilizing purposive sampling. We will approach staff who have had particularly interesting or meaningful experiences as a result of their engagement with the study and treatment modality of telemedicine.

Administrators

We will plan to interview all administrators – one to two, depending on a site or organization.

For the staff members, the case manager will also contact them either in person, or phone or by email.

Non-subjects

Patients at the clinics who were invited to be part of the study but declined to participate and are still going to the same clinic may be approached during their clinic visit with an invitation to be interviewed. Eligible patients will be found based on screening records from the main study or study staff recollections.

*9.2 Describe how you will protect the privacy interests of prospective subjects during the recruitment process.*

*NOTE: Privacy refers to an individual’s right to control access to him or herself.*

Response: Protection against privacy breach by participants will be ensured by discussions of study enrollment done in a private setting, between the study case manager at each participating site and the potential study participant. Similarly, for patients in the telemedicine arm, HCV therapies will be dispensed at the methadone dispensing window at the same time as methadone to minimize the chance that other patients from the OAT program might observe a study subject taking HCV medications. As part of the informed consent, we will also request consent from patients to contact their medical providers and HCV specialists in order to follow up on the referral requests to determine if the patient actually went for the referral from their primary provider to an off-site Liver specialist.

Data security. Stored data protections will be designed in collaboration with the Information Technology Departments at each of the participating institutions as well as with the data management systems, My Own Med (MoM) and Breadcrumb Analytics, we will employ for this protocol. Protections employed in this study will be similar to those that have been enacted in similar clinical research studies. Data will be stored on password protected servers. Patient identity will not be shared with anyone other than the staff at participating sites and the physicians who are treating the patients. Each participating site will be assigned a site number, with each patient provided a single study identification number in ascending order by the study coordinator for all study related data pertaining to the subject. This information once de-identified will be stored additionally on a data management server housed at the Institute for Healthcare Informatics or similar university-designated server at the University at Buffalo and monitored by study staff at UB Research. Additionally, in all participating OAT clinics, all study personnel will be trained and certified in human subject studies protection. Furthermore, training in human subject’s protection and research methodology will be part of the engagement process for any study related procedures to be conducted as part of this protocol.

*9.3 Identify any materials that will be used to recruit subjects.*

*NOTE: Examples include scripts for telephone calls, in person announcements / presentations, email invitations.*


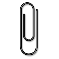
*For advertisements, include the final copy of printed advertisements with your submission. When advertisements are taped for broadcast, attach the final*

*audio/video tape. NOTE: You may submit the wording of the advertisement prior to taping to ensure there will be no IRB-required revisions, provided the IRB also reviews and approves the final version.*

Response: At present, we will not rely on advertisements or video production but will recruit people from the electronic health record or through the screening process described in sect.7.1 Eligibility Screening

## 10.0 Procedures Involved

*10.1 Provide a description of* ***all research procedures or activities*** *being performed and when they are performed once a subject is screened and determined to be eligible. Provide as much detail as possible.*

*NOTE: This should serve as a blueprint for your study and include enough detail so that another investigator could pick up your protocol and replicate the research. For studies that have multiple or complex visits or procedures, consider the addition of a schedule of events table in in your response.*

Response: All participating OAT clinics will screen patients for HCV Antibody as required by New York State. At each participating OAT clinic, the designated study manager will meet privately with each patient identified as HCV Antibody positive to discuss the study. Once consented, blood specimens would be obtained (up to 44 ml) to determine HCV RNA, geno-typing, Drug sensitivity, HIV RNA, CD4 count (to be offered if in not in patient’s chart), HBsAb, HBsAg, complete blood count, comprehensive metabolic panel with estimated glomerular filtration rate, international normalized ratio, prothrombin time, and a referral either directly to a specialist or to their primary care physician (if HCV RNA detected). Once a referral has been issued, the designated study case manager of the OAT clinic will interact with the patient on a monthly basis to determine whether they have complied with the referral as well as medication adherence. HIPAA consent and contact information of the primary care physician as well as the liver specialist will be obtained. In the case of one-step referral process, the coordinator will be able to follow up directly with the liver specialist. In the case of a two-step referral process, the coordinator will follow up with both the primary care provider as well as the liver specialist. Some payers may require the two-step process as they may require that all referrals be completed by the PCP. We will administer the Hepatitis C Patient Satisfaction Questionnaire, Modified Mini Mental Health Screen, National Institute on Drug Abuse Quick Screen, Drug Abuse Screening Test (DAST), and Sociodemographic Survey to patients who are referred for HCV management at an offsite location. Similarly, in both arms, subjects will be given a standardized brochure about HCV produced by SAMHSA.

After 9 months, 4 randomly selected clinics will begin consenting and enrolling patients into the telemedicine arm. This randomization will continue every 9 months until all clinics have been transitioned into the telemedicine arm. Screening and consent will be conducted in the same manner described for the referral arm. We will offer HCV-related interactive educational sessions to the patients enrolled in telemedicine treatment of HCV at each clinic during its telemedicine period. These sessions will cover the specifics of telemedicine as a means for delivery of healthcare and will address the concerns, such as privacy and confidentiality. During the telemedicine visits, patients will be linked with the provider via two-way videoteleconferencing, which may be, depending upon a visit, facilitated by an onsite OAT program staff member. The educational sessions will also include the principles of telemedicine as well as an introduction, including the credentials of the physician conducting telemedicine-based appointments and the privacy of medical data. Family members and patient caregivers will be invited to participate in the initial session so that they may be a means of moral support to the patient during HCV treatment. During this appointment, patients will be evaluated for liver fibrosis that only requires a routine blood sample (Fibrosure)

Two weeks later, patients will have a *second telemedicine appointment* when their results will be discussed with the liver specialist. If the patient meets treatment eligibility criteria and is willing to undergo HCV therapy, medications will be prescribed using the standard format requested by our specialty pharmacy partner. This telemedicine visit will be concluded with specific information provided to the patient concerning the medication regimen to be followed and side effects. The patient will then be instructed on how to take the HCV medications and the importance of adherence to the HCV treatment regimen will be emphasized. HCV medication will be administered as directly observed therapy (DOT) dispensed through the methadone dispensing counter in the OAT program. Subjects in both arms will be given Hepatitis C Treatment Satisfaction Questionnaires at the time of the first visit, and three months post treatment cessation.

In the Telemedicine arm, HCV RNA will be obtained at baseline, treatment week 2, treatment week 6, at the end of treatment, post treatment week 4, and at 12 weeks posttreatment cessation. The blood will also be obtained at 6, 12, 18, and 24 months posttreatment to monitor for HCV reinfection. Follow up tests for reinfection may be done by standard blood draw or by dry blood spot testing, which involves a finger prick.

DBS (dried blood spot) testing is used to assess HCV RNA (Hep C viral load). If the test comes back positive, we would confirm HCV RNA with venipuncture. At the same time, we would likely check the genotype for genotype switching. A different genotype would likely indicate reinfection.

All telemedicine appointments will occur online with the liver specialist. The OAT Program staff clinician (MD/NP/PA) will be requested to be present based on the clinical need (e.g., non-compliance with therapeutic regimen, issues related to substance use). Telemedicine appointments will occur at the initial evaluation, when the HCV medication is ordered, and 2 weeks into treatment. Post-treatment, telemedicine appointment will occur at week 12. Other visits are optional and are based on the clinical need. Counseling sessions aimed at preventing reinfection will be offered to both arms at post treatment week 12 visit. Finally, we will administer the Hepatitis C Patient Satisfaction Questionnaire, Drug abuse Screening Test three months post treatment cessation. Among patients in both arms who achieve viral eradication, HCV RNA, toxicology screens will be obtained at week 4 post treatment, week 12 post treatment and months 6, 12, 18 and 24 post treatment cessation. This blood may be shipped from the clinic to SUNY at Buffalo, where it may be assayed for HCV RNA and HCV genotyping. We may also store an aliquot of blood for future reference including sequencing for new viruses. We may also decide to sequence the baseline virus to investigate for viral genetic diversity.

If a patient is found to again test positive for HCV during their follow up period, we will collect data about their new infection, and draw up to 3 tbsp of blood at 2 and 6 weeks after the beginning of retreatment, at the end of treatment and at 4 and 12 weeks after the end of treatment. We may also decide to sequence samples obtained from any patients who have a positive HCV RNA sample after successful viral eradication in order to differentiate those who have reinfection versus relapse. We may also measure the HCV core antigen on these samples.

Collecting of HCV reinfection data occurs only during the subject’s study period, that is, up to 24 months from the end of their first study-related HCV treatment. If the subject is not retreated, we will continue monitoring him/her for a potential treatment start date during the study period and if s/he starts new treatment, we will collect follow-up data until the end of their study period. If the subject is reinfected at the end of his/her study period, i.e., Visit 11 in the Usual Care Arm and Visit 12 in the Telemedicine Arm of the study, there will be no further data collection.

For the collection of social determinants of health (SDOH) data: Information from the subject’s initial intake forms (local to each site: Comprehensive assessments), as well as any follow-up assessments done in subsequent years, will be entered into a data collection spreadsheet.

Then the PAS-44 and certain PAS-26s will be collected as follows:

1. Following instructions sent from OASAS, data from the PAS-44, administered at patient’s admission to the opioid treatment program, and specified PAS-26s, will be downloaded into an Excel spreadsheet from OASAS’s website (this is a secure download through their system). The data will be extracted by a member of the study team.
2. The spreadsheet is generated by OASAS’s program based on entering a date range of up to a year. A spreadsheet is created that contains the information for all the clinic’s patients that filled out the PAS form for that date range.
3. After the spreadsheet is created, without opening the file, it will be dropped into a computer program created by Breadcrumb Analytics that has been downloaded onto the local computer. The program will allow the user to enter a set of patient identifiers

(which will include some of the following identifiable data points: Provider Client ID, Birth Date, Last 4 SSN, Last Name-First 2 Letters, First Name-First 2 Letters) to select only the data related to the study subjects. The program will extract the data for the selected patients, and remove the identifying columns (All data points in TRS61 and TRS-49 sections,, please see screenshot below). It will also change the date of admission and date of assessment completion to just month and year. All other records of non-study subjects will be discarded and deleted.

1. The program will then allow the user to inspect the other fields to verify that there is no identifying information. Once the records have been verified as containing no identifiable information except for the Study ID#, the program will allow the user to send the files to the UB study team over secure FTP.
2. The time sequence of requested PAS-26 is as follows:

The first one administered (that is, the first annual update), 4 years prior to the study, and every year after that until the participant completes the study or is otherwise discontinued from the study (up to approximately 10 PAS-26).


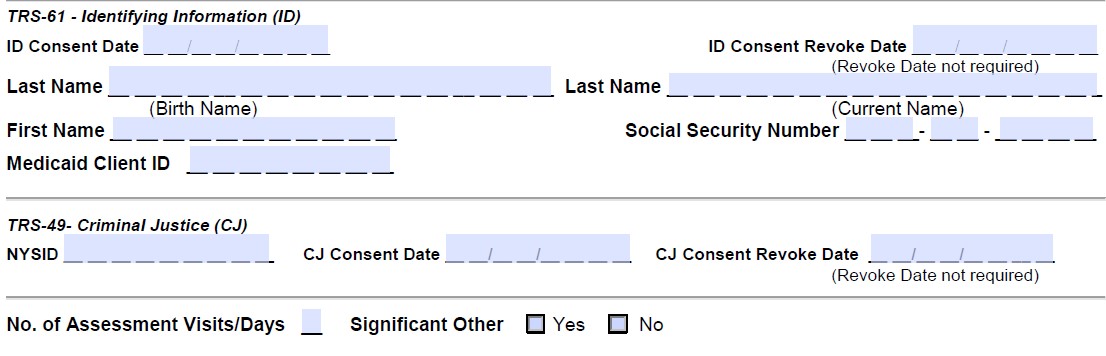


: Patient Advisory Committee: Patient Advisory Committee members and study participants may be requested to speak on behalf of their experiences participating in the study, their experiences with HCV treatment, or on the dissemination of results of the study. These appearances may consist of media interviews, one-on-one interviews, or public speaking engagements.

The purpose of these interviews and speaking engagements is not for recruitment into the study. The purpose of the dissemination of results is to spread the word about the benefits of telemedicine to a wide variety of stakeholders including patients, clinic staff, and insurance companies. The goal is to familiarize patients with the procedures, to encourage financial sustainability through appropriate telemedicine reimbursement, and to promote the growth of the modality as a method to deliver healthcare.

Interview substudy:

Some of the subjects from the parent study will be invited to be interviewed about their experiences, opinions and feelings about the study, medical care in general, and their OTP clinic and community. The interviews will take place over videoconferencing, the phone, or in person. All interviews will be recorded and transcribed.

Subjects will be interviewed by someone on the study staff who does not work in the subject’s OTP.

The questions will fall under the following general categories:

- Trust in the OTP clinic and community (Example: How would you describe your relationship with clinic staff, i.e., counselor, physician?)
- Experience with the research study (Examples: What attracted you to being in the PCORI study? Features that made you skeptical about being in the study?)
- Challenges with receiving medical care outside of the study (Example: What are the barriers or problems you do have or have encountered in general medical settings?)
- Experiences with personalized care in the OTP (Example: What are some of the helpful things that clinic staff does to manage your treatment plan and prioritize your needs (including addiction, comorbidities, mental health issues, insurance/finances, transportation)?)

Some of the staff who have been working at the clinic during the conduct of the study will be interviewed, about their feelings, opinions and experiences with telemedicine in general, as well as specifically their observations about the use of telemedicine to implement HCV treatment.

Interview guides are included in this submission.

Contacts at sites that did not choose to participate in the parent study will be interviewed about the reasons why and their opinion about telemedicine.

Patients at the clinics who were invited to participate in the main study and declined and agree to be interviewed will participate in a single interview either by Zoom, telephone, or in person. They will be asked questions in the following categories:

- - 1. Why did you decide not to pursue HCV treatment and/or participate in the study?
    2. Is there anything that we could do to get you ready for treatment?
    3. Are there any areas where you think education or knowledge could be helpful?
    4. Do you have any other recommendations for us?

|  |
| --- |

|  |
| --- |

*10.2 Describe what data will be collected.*

*NOTE: For studies with multiple data collection points or long-term follow up, consider the addition of a schedule or table in your response.*

Response: The following data will be collected from patients enrolled in the study:

- - 1. Demographic information (age, sex, race, ethnicity)
    2. Height
    3. Weight
    4. Body Mass Index
    5. Marital status
    6. Type of residence
    7. Employment status
    8. Legally disabled
    9. Highest level of educational attainment
    10. Type of insurance
    11. Co-morbid medical and psychiatric diagnoses
    12. Medications with current dose
    13. CD4 cell count and HIV RNA level on patients chart- if status unknown HIV testing to be offered
    14. Data related to HCV including HCV RNA, HCV genotype, fibrosis level, inflammatory grade, HCV resistance associated variants, complete blood count, comprehensive metabolic panel with estimated glomerular filtration rate, international normalization ratio, and prothrombin
    15. Data related to hepatitis B virus infection-HBV DNA level, HBsAb, HBsAg
    16. Opiate agonist specific variables-methadone peak and trough levels, type of treatment, dose and duration.
    17. Illicit drug use and behavior
    18. For sites that have access to FibroScan, we will collect fibrosis measurements and steatosis level.
    19. Model of end-stage liver disease (MELD)
    20. Drug Abuse Screening Test (DAST)
    21. Billing and claims data such as CPT code, gross charges, payment, patient cost share (co-pay or co-insurance), reason for denial of payment by the insurance company.

|  |
| --- |

- - 1. Patient stories and events will be collected from study case managers and study staff to illustrate how the telemedicine model with accompanying medication dispensing facilitates HCV treatment. No identifiable patient information will be included.

Information to be collected includes patient-reported outcomes, such as how the onsite treatment delivery facilitated treatment completion, overcoming stigma barriers, obtaining secure housing in a homeless shelter, and obtaining treatment in the case of physical immobility.

| 23. Social determinants of health – behavioral, environmental, and demographic characteristics that can affect a patient’s health. Please see Comprehensive Assessment and SDOH in  Supporting Documents. These may be from the versions done at the subject’s initial admission to the clinic, or during follow up administrations of the same and/or similar forms over the time since the initial admission. We will also collect the data from the PAS-44 and PAS-26s (see section 10.1 for schedule) minus the identifiable fields. The PAS forms are the SDOH variables collected by OASAS from each clinic, and overlap the local clinic’s forms. |
| --- |

Interview substudy:

General demographics from staff will be collected (age, gender, ethnicity), job title, and degrees received. (See data collection instrument). Everything else that will be collected from both patients and staff will be qualitative data such as opinions and experiences from recorded and transcribed interviews and focus groups. No quantitative data will be collected from patients as all required quantitative data have been collected as part of the parent study.

General demographics will be collected from the non-participants as well – age, gender, ethnicity and educational level completed.

|  |  |
| --- | --- |
|  | |

*10.3 List any instruments or measurement tools used to collect data (e.g.*
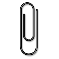
 *questionnaire, interview guide, validated instrument, data collection form).*

*Include copies of these documents with your submission.*

Response: All participants will complete a Modified Mini Mental Health Screen, National Institute Drug Abuse Quick Screen and Sociodemographic Survey at the first study visit. We will also administer the Drug Abuse Screening Test (DAST) at screening, and Patient Satisfaction Questionnaire at 3 months post treatment cessation.

If a subject is found to be reinfected with HCV, the information about the new treatment episode will be collected on an Excel spreadsheet (attached).

Interview substudy:

Videoconference recordings and transcripts or digital voice recordings and transcripts will be used to collect the interview and focus group data. Interview guides are included in this submission. Please see attached Excel file for the quantitative data to be collected from staff.

*Describe any source records that will be used to collect data about subjects (e.g. school records, electronic medical records).*

Response: Data will be collected from Electronic Medical Records using an electronic file transfer through MoM. Additionally this de-identified information will be stored on a data management server, housed at the Institute for Healthcare

Informatics or similar university-designated server at the University at Buffalo and monitored by study staff at UB Research. Financial data for the telemedicine subjects and usual care subjects that were treated by the PI will be extracted from the UBMD or START financial reports that the PI receives as the rendering provider.

Financial data for usual care subjects not treated by the PI will be requested from subjects’ insurance companies or treating physician/practice.

Social Determinants of Health may be accessed in paper medical records if the information has not been added to the clinic’s EMR. The PAS-44 and PAS-26 will be downloaded from the OASAS servers through an online portal at each clinic.

Interview substudy: NA

|  |
| --- |

*10.4 Indicate whether or not* ***individual*** *subject results, such as results of investigational diagnostic tests, genetic tests, or incidental findings will be shared with subjects or others (e.g., the subject’s primary care physician) and if so, describe how these will be shared.*

Response: Each subject will be privately given all test results at screening and at each visit. Consents will be obtained to share collected information with primary physicians and/or liver specialists.

*10.5 Indicate whether or not* ***study*** *results will be shared with subjects or others, and if so, describe how these will be shared.*

Response: Presentations will be done at participating clinics in order to share our findings with study participants as well as with other patients who might be interested in the study results. Similar presentations will also be organized in other methadone clinics. We plan to engage the Patient Partners (a group of patient volunteers who have gone through HCV treatment via telemedicine) in the dissemination of the results within their own clinics and to outlaying clinics. Many patients seen in these clinics are HCV infected and may be very interested in the study findings and embrace alternative strategies for HCV management and treatment other than usual care, referral to an offsite location. Results of this study will be made available on ClinicalTrials.gov

Information collected from study case managers and other study staff (see 10.2 #23) will be used to create a manuscript of interesting patient stories or events to illustrate how the telemedicine model with accompanying medication dispensing facilitates HCV treatment. We have had a variety of instances where patients have reported that the model directly or indirectly facilitated their overcoming obstacles to healthcare access and delivery related to HCV. The manuscript will be an opportunity to capture these events to illustrate ancillary benefits of integrated addiction and HCV treatment in our telemedicine model.

Study results will be shared with stakeholders at the national, state, and local levels to obtain their input and perspectives for drafting a variety of manuscripts based on the study. These stakeholders, who may or may not have been involved in the implementation and conduct of the study, may include patient-participants, case managers, and clinic administrators and staff. No identifiable information will be shared.

## 11.0 Study Timelines

*11.1 Describe the anticipated duration needed to enroll all study subjects.*

Response: The anticipated duration to enroll all study subjects is 36 months.

Interview substudy:

3 months for patients, staff 6 months.

*11.2 Describe the duration of an individual subject’s participation in the study. Include length of study visits, and overall study follow-up time.*

| Response: Subjects will be enrolled for approximately 32 months in the study. This would encompass time of initial screen to the last visit 2 years post treatment.  Each visit is expected to require an average of 1 hour of subject’s time.Interview substudy:  Interviews will take about an hour. PCORI subjects and staff will be interviewed once, and some may be invited to participate in a one time focus group, which would also take about an hour. Non-participants, i.e., individuals who declined to participate in the parent study, will be invited to participate in a one-time interview. |
| --- |

*11.3 Describe the estimated duration for the investigators to complete this study (i.e. all data is collected and all analyses have been completed).*

Response: The estimated time for the investigator to complete the study is 5.5 years.

Interview substudy:

Approximately 6 months to collect the data and an additional 6 months to analyze it.

## 12.0 Setting

*12.1 Describe all facilities/sites where you will be conducting research procedures. Include a description of the security and privacy of the facilities (e.g. locked facility, limited access, privacy barriers). Facility, department, and type of room are relevant. Do not abbreviate facility names.*

*NOTE: Examples of acceptable response may be: A classroom setting in the Department of Psychology equipped with a computer with relevant survey administration software,” “The angiogram suite at Buffalo General Medical Center, a fully accredited tertiary care institution within New York State with badge access,” or, “Community Center meeting hall.”*

Response: All participating OAT clinics must provide a patient-friendly, private examination room for discussion of HCV detection, study information, and consenting. For telemedicine they must employ two-way, face to face communication using a HIPAA- compliant, secure web – connected computer equipment with digital camera.

Interview substudy:

Interviews will happen over the phone, over videoconferencing, or in a private room in the OTP clinic.

*12.2 For research conducted outside of UB and its affiliates, describe:*

- *Site-specific regulations or customs affecting the research*
- *Local scientific and ethical review structure*

*NOTE: This question is referring to UB affiliated research taking place outside UB, i.e. research conducted in the community, school-based research, international research, etc. It is not referring to multi-site research. UB affiliated institutions include Kaleida Health, ECMC, and Roswell Park Cancer Institute.*

Response: Submission, review and approval of the protocol will be at the local

IRB level. Those sites without a local IRB sites 01 and 11, will follow UB-IRB guidelines and approvals. All sites will meet state regulations and licensing requirements for treatment of substance abuse patients. UB is acting as the main coordinating site. All the other sites will submit their clinical data to UB through MyOwnMed (MoM), and the billing data and SDOH data through other secure means. There is no data sharing between sites laterally. Data Use Agreements are in place between UB and the participating sites.

☐ **N/A:** This study is not conducted outside of UB or its affiliates.

## 13.0 Community-Based Participatory Research

*13.1 Describe involvement of the community in the design and conduct of the research.*

*NOTE: Community-Based Participatory Research (CBPR) is a collaborative approach to research that equitably involves all partners in the research process and recognizes the unique strengths that each brings. CBPR begins with a research topic of importance to the community, has the aim of combining knowledge with action and achieving social change to improve health outcomes and eliminate health disparities.*

Response: This research will be conducted in a largely medically disenfranchised, largely under-represented minority group of patients in substance use treatment. In addition to protocol reviews by local IRBs, a patient-partner group led by a patient advocacy manager, will meet every month or quarterly to discuss the details of treatment practice and to provide feedback on the study conduct to share with participating sites and within their own communities. As this study is supported by the Patient-Centered Outcomes Research Institute (PCORI), patient and community engagement of interested stakeholders is a crucial part of the entire study. Meetings will occur at least annually with patient and stakeholder advisory groups where feedback will be ascertained on mechanism by which to improve the study conduct.

☐ **N/A:** This study does not utilize CBPR.

*13.2 Describe the composition and involvement of a community advisory board.*

Response: For this study, the “community advisory board” will be comprised of two separate committees, a patient advisory board and a sustainability committee comprised of stakeholders. The objective of each will be to educate and to advocate for treatment and support services for individuals with viral hepatitis. Patient partner groups will consist of a patient advocate acting as facilitator and 3 patients who have completed or who are undergoing HCV treatment at a participating site. The Sustainability Committee will be comprised of representatives of New York State and City Departments of Health, leading pharmaceutical manufacturers of HCV treatments and diagnostic companies, third party payers, HCV educational foundations, medical providers, telemedicine implementation expert, and federal agencies involved in treatment of patients with substance use disorders.

☐ **N/A:** This study does not have a community advisory board.

## 14.0 Resources and Qualifications

*14.1 Describe the qualifications (e.g., education, training, experience, expertise, or certifications) of the Principal Investigator* ***and*** *staff to perform the research. When applicable describe their knowledge of the local study sites, culture, and society. Provide enough information to convince the IRB that you have qualified staff for the proposed research.*

*NOTE: If you specify a person by name, a change to that person will require prior approval by the IRB. If you specify a person by role (e.g., coordinator, research assistant, co-investigator, or pharmacist), a change to that person will not usually require prior approval by the IRB, provided that the person meets the qualifications described to fulfill their roles.*

Response: The Principal Investigator (PI) of this study, Dr. Andrew Talal is a board-certified gastroenterologist who has been treating patients with HCV infection for the past 25 years, and who has been conducting a pilot study of HCV treatment via telemedicine for the past 5 years at one of the participating sites (START Treatment and Recovery Centers). At the European Association for the Study of Liver Diseases annual meeting in April 2016, Dr. Talal illustrated the results of the first 19 methadone-maintained patients treated for HCV via telemedicine that showed excellent efficacy, medication adherence, and acceptance of the modality. The research teams, at each participating site consist of experts in addiction medicine, infectious diseases, implementation science, epidemiology, biostatistics, and addiction psychiatry. All will be responsible for general study oversight as delegated and supervised closely by the PI.

Selected study team members will be invited to participate in focus groups, dissemination workshops, data acquisition/analysis activities, and manuscript revision/approval processes. All data will be de-identified before sharing with these individuals. These individuals will serve as sub-study participants and may also be authors on publications.

***Describe other resources available to conduct the research.***

*14.2 Describe the time and effort that the Principal Investigator and research staff will devote to conducting and completing the research.*

*NOTE: Examples include the percentage of Full Time Equivalents (FTE), hours per week. The question will elicit whether there are appropriate resources to conduct the research. Response:*

**Principal Investigator (20% Effort each year)**

**Co-Investigator: (10% Effort Y1-4, 5% Effort Y5).**

**Co-Investigator: (5% Effort Y1 and 5, 10% Effort Y2-4).**

**Biostatistician: (20% Effort Y1, 10% Y2-3, 30% Y4-5)**

**TBN Project Coordinator/Managed care coordinator:**

**(50% Effort Y1-4, 25% Y5)**

**TBN - Recruiter:**

**(50% Effort Y1-4)**

**TBN - Telehealth IT support:**

**(20% Effort Y1-3, 10% Y4)**

**TBN - Clinician trainer:**

### (40% Effort Y1-4, 20% Effort Y5)

|  |
| --- |

*14.3 Describe the availability of medical or psychological resources that subjects might need as a result of anticipated consequences of the human research, if applicable.*

*NOTE: One example includes: on-call availability of a counselor or psychologist for a study that screens subjects for depression.*

Response: The study will be conducted at sites that have familiarity with the treatment of mental health disorders in patients in substance use treatment as well as 24-hour medical coverage for emergencies. Secondly, in the case that a particular site does not have the requisite expertise to address a medical issue, each participating site could contact other sites that could support patient management in specific areas that may be lacking at the individual clinic. Thirdly, each individual site study staff will be available 24 hours a day, 7 days a week to address issues that may not be readily addressed by the above mentioned methods.

*14.4 Describe your process to ensure that all persons assisting with the research are adequately informed about the protocol, the research procedures, and their duties and functions.*

Response: Clinicians, staff, and administrators from the participating OAT clinics will be engaged at the outset of the project by convening multiple information and staff training sessions given by UB Research staff to introduce the study, to explain the rationale and background for the initiative, and to review the protocol. Update meetings will be conducted to share progress and to address challenges.

## 15.0 Other Approvals

*15.1 Describe any approvals that will be obtained prior to commencing the research (e.g., school, external site, funding agency, laboratory, radiation safety, or biosafety).*

Response: Participating OAT sites must have all state required licenses prior to consideration of participating in research study. Protocols will be submitted, reviewed and approved by local IRBs. Those sites without a local IRB will use UB-IRB approved protocol. Physicians delivering telemedicine must be licensed in the state where services are delivered and versed in the state and payer-specific reimbursement processes. The sponsor, PCORI, has already approved the study concept.

Interview substudy:

All study sites not depending on UB IRB will obtain IRB approval at their institutions.

☐ **N/A:** This study does not require any other approvals.

## 16.0 Provisions to Protect the Privacy Interests of Subjects

*16.1 Describe how you will protect subjects’ privacy interests during the course of this research. NOTE: Privacy refers to an individual’s right to control access to him or herself. Privacy applies to the person. Confidentiality refers to how data collected about individuals for the research will be protected by the researcher from release. Confidentiality applies to the data.*

*Examples of appropriate responses include: “participant only meets with a study coordinator in a classroom setting where no one can overhear”, or “the participant is reminded that they are free to refuse to answer any questions that they do not feel comfortable answering.”*

Response: During visits, clinicians and specialist will meet with patients in private, comfortable, closed examination rooms to discuss lab results, plans of care and progress.

Subjects who agree to be interviewed in an external setting for study dissemination activities will sign a separate consent form and will be asked about each interview opportunity separately. Alternatively, their participation will be affirmed by certification that they agree to be coauthors on publications resulting from this investigation. These subjects will be reminded that any information that they share, as well as their status as active participants in a study about hepatitis C treatment conducted in an OAT clinic, will no longer be private. These individuals will serve as sub-study participants; they are not methadone patients.

Interview substudy:

During videoconference or phone interviews, privacy on both sides of the call will be ensured, with the interview being rescheduled if privacy is not possible. Participants will be reminded that they are free to refuse to answer any questions that they do not feel comfortable answering.

*16.2 Indicate how the research team is permitted to access any sources of information about the subjects.*

*NOTE: Examples of appropriate responses include: school permission for review of records, consent of the subject, HIPAA waiver. This question* ***does apply*** *to records reviews.*

Response: Subjects will be asked to sign a HIPAA consent and a study-specific informed consent to allow primary providers, liver specialists, and their particular substance use treatment site to share patient information pertaining to treatment of HCV and as required for the conduct of the study. A Partial HIPAA Waiver will be submitted at each site for review of potential subject’s medical records at screening.

A HIPAA waiver is being requested to analyze the billing data for the subjects in the telemedicine arm and those in the usual care arm who were treated by the PI.

Modification 5/8/2020 - A HIPAA waiver is also being requested to collect the billing data for the usual care arm subjects who were treated elsewhere, by requesting the billing data from the insurance companies or from the treating physician.

A HIPAA Waiver is also being requested to collect retrospective data from the subject’s clinic records and the OASAS server on their SDOH. This will apply to the subjects from the two sites covered by UB-IRB only. All other sites will submit to their own IRBs.

## 17.0 Data Management and Analysis

*17.1 Describe the data analysis plan, including any statistical procedures. This section applies to both quantitative and qualitative analysis.*

*Response:* In this study, we will implement a stepped wedge cluster randomized controlled trial with two arms (intervention and control). The statistical analysis will be performed using SAS (SAS Institute Inc., Cary, NC, USA) and R ([http://www.r-project.org/)](http://www.r-project.org/). The data collection will be conducted over 4 time points with 3 steps with 4 clinics switching (in a random order) from control to intervention at each step. The analysis set will include all patients who satisfy the patient level eligibility criteria (6 months active OTP enrollment, HCV RNA positivity, and medical insurance; exclusion criteria are HCV medication contraindications and HCV treatment elsewhere) and consent to participate.

We will follow the intention to treat principle. Enrolled subjects’ characteristics will be summarized by their arm and by time period. In addition to demographic data (sex, age, race, ethnicity, education, employment), we will also collect data on opiate agonist therapy-specific variables (type of treatment, duration, therapeutic regimen), illicit drug use behavior (frequency, abstinence), HCV-specific variables (HCV RNA, HCV genotype, fibrosis, ALT, AST, hemoglobin, platelet count, sodium, total bilirubin, creatinine, estimated glomerular filtration rate, prothrombin, international normalized ratio, previous HCV treatment) and co-morbidities (HIV, HBV, psychiatric). Continuous variables will be presented by their mean (and standard deviation) and median (and interquartile range [IQR]). Categorical variables will be presented by their frequencies. We will assess comparability between the two arms. Categorical variables will be compared through Fisher’s exact test, and continuous variables will be compared through Wilcoxon’s ranksum test.

The primary outcome of the study is achievement of SVR, which is defined as undetectable HCV RNA 12 weeks post treatment cessation. For the primary analysis, the SVR rate will be evaluated based on all study patients (HCV RNA+, age ≥18, at least 6months of OTP, insurance coverage) who consent to referral (usual) care or telemedicine management of HCV. Primary analysis will be individual-level based with binary outcome, corresponding to successful treatment outcome (achievement of SVR), using GLMM or GEE modelling to account for the clustering. The GLMM model will include a random effect for each cluster, as well as a fixed effect for each time period and for the cluster (clinic) intervention assignment at each time point. We will check for interaction between time and intervention and will adjust as necessary for covariates to account for potential differences between the two arms. The intervention effect will be expressed as odds ratio and will be reported together with its 95% confidence interval and p-value, and both unadjusted and adjusted odds ratios will be reported. Model assumptions will be checked at every level of the analysis through graphical and analytical tools. Additionally, for patients assigned to the telemedicine arm who do not adhere to at least one telemedicine appointment, we will also account, by subgroup analysis, for the actual management strategy for HCV treatment that each patient utilized (i.e., whether at least one telemedicine appointment was completed).

As secondary outcomes, we will collect information on Hepatitis C patient satisfaction surveys, National Institute on Drug Abuse Screen, Modified Mini Mental Health Screen, Drug Abuse Screening Test and Sociodemographic Survey, treatment initiation rate, treatment adherence rate, treatment completion rate and HCV reinfection rate at the end of the study. We will assess for the questionnaire’s internal consistency by calculating the Cronbach’s alpha. We will compare patient satisfaction with the delivery of health care between the two arms using linear mixed effects model or GEE. For the comparison of the treatment initiation and treatment completion rates between the two arms, we will apply the same methodology as for the analysis of the SVR rate (above). Adjustment for covariates will be incorporated as appropriate. HCV reinfection will be represented by a binary variable indicated as HCV RNA positivity and will be modeled using GLMM or GEE. All tests will be two sided with level of significance of 0.05. Most covariates for adjustment of the analyses for potential confounding will be assessed during the screening process of the study either directly from the patient or from the patients’ medical record. We will use graphical and analytical methods for detection of outliers in the data and conduct sensitivity analysis by fitting the model with and without the suspicious observations. The suspicious observations will be checked for accuracy.

We will be evaluating the resource utilization used in treating Hepatitis C via telemedicine as a way to minimize the long term complications of the virus, such as liver transplants, liver cancer and cirrhosis. Resource utilization will be analyzed for regional differences and other socioeconomic variables.

SDOH will be analyzed for any association with the pursuit of HCV treatment. For the analysis of SDOH data, we may use large language models provided by Open AI using their application programming interface (API) to pre-process the data for statistical analysis. We will abide by Open AI’s procedures for processing data following their terms, conditions, and protocols. The UB Purchasing Department and the Office of Medical Computing are carefully reviewing the terms and conditions associated with procuring the Open AI large language model API access. Terms and Policies associated with the program are located at <https://openai.com/policies>. Policies related to an addendum for Personal Data Processing is located at <https://openai.com/policies/data-processing-addendum>. The following form will be completed as required for compliance with the personal data processing: Data Processing Addendum Form -<https://ironcladapp.com/public-launch/63ffefa2bed6885f4536d0fe>.

We may also utilize the language models provided by Google, and Meta, in addition to Open AI, to aid in data processing for further analyses. We may employ Google's PaLM 2 language model to perform various data processing tasks. This language model can be accessed through the PaLM API, which is also a part of the Vertex AI platform on Google Cloud.

Google

Product link - https://developers.generativeai.google/products/palm

The associated terms and conditions can be found at -

https://policies.google.com/terms/generative-ai

https://developers.google.com/terms

https://cloud.google.com/terms/service-terms

https://cloud.google.com/security/compliance/

https://cloud.google.com/ai/generative-ai

Meta

The large language model from Meta is called Llama 2. It is another language model that we might use for performing data processing tasks. This language model can be downloaded from Meta and setup on a local machine without the need to use API to interact with the model.

The terms and conditions can be found here at -

[https://ai.meta.com/resources/models-and-libraries/llama-downloads/](https://nam12.safelinks.protection.outlook.com/?url=https%3A%2F%2Fai.meta.com%2Fresources%2Fmodels-and-libraries%2Fllama-downloads%2F&data=05%7C01%7Cahtalal%40buffalo.edu%7C2a37520464454f54f87b08dbda127e61%7C96464a8af8ed40b199e25f6b50a20250%7C0%7C0%7C638343545182663922%7CUnknown%7CTWFpbGZsb3d8eyJWIjoiMC4wLjAwMDAiLCJQIjoiV2luMzIiLCJBTiI6Ik1haWwiLCJXVCI6Mn0%3D%7C3000%7C%7C%7C&sdata=LWw4Q6V%2BwtZjnLW48F9H%2FfFNsv0BwN8DK7qAk%2BJluZ4%3D&reserved=0)

[https://ai.meta.com/llama/use-policy/](https://nam12.safelinks.protection.outlook.com/?url=https%3A%2F%2Fai.meta.com%2Fllama%2Fuse-policy%2F&data=05%7C01%7Cahtalal%40buffalo.edu%7C2a37520464454f54f87b08dbda127e61%7C96464a8af8ed40b199e25f6b50a20250%7C0%7C0%7C638343545182663922%7CUnknown%7CTWFpbGZsb3d8eyJWIjoiMC4wLjAwMDAiLCJQIjoiV2luMzIiLCJBTiI6Ik1haWwiLCJXVCI6Mn0%3D%7C3000%7C%7C%7C&sdata=XW6n%2BdqPkrMtXfv30z3Wr%2FynNi%2BWYOycG49ZsfnYzxk%3D&reserved=0)

We are also working with OMC, the VPRCIO’s office, and purchasing for the approval of these large language models.

Interview substudy:

Topic areas of interest for both patients and staff will be developed by a group of experts in the area of qualitative research including a clinical psychologist with significant experience with substance use research in the criminal justice system, a UB Professor of Nursing, and an Infectious Diseases physician who has conducted research in OTPs for the past two decades. Combined with experienced case managers and the PI of the parent study, we will develop interview/focus group/survey topics to be assessed. We anticipate asking these topics in sections...

For qualitative research, we will analyze the data using Hermeneutic phenomenology to interpret and to explicate common meanings and shared practices of the phenomena.

*17.2 If applicable, provide a power analysis.*

*NOTE: This may not apply to certain types of studies, including chart/records reviews, survey studies, or observational studies. This question is asked to elicit whether the investigator has an adequate sample size to achieve the study objectives and justify a conclusion.*

Response: Please see below for the sample size calculations for the primary (Table 1) and secondary (reinfection) (Table 2) endpoints.

**Table 1. Effect Size, Sample Size and Statistical Power for Primary Aim of Viral**

**Eradication**

| **Telemedicine Arm** | |  | **Referral Arm (Usual Care)** | | | **80% power** | **90% power** |
| --- | --- | --- | --- | --- | --- | --- | --- |
| **Treatment**  **Initiation** | **SVR^a^** | **SVR^b^** | **Treatment**  **Initiation** | **SVR^a^** | **SVR^b^** | **N**  **Total** | **N**  **Total** |
| 25% | 80% | 20% | 15% | 80% | 12% | 518 | 681 |
| 30% | 80% | 24% | 15% | 80% | 12% | 257 | 336 |
| 30% | 80% | 24% | 18% | 80% | 14.4% | 417 | 548 |
| 30% | 80% | 24% | 20% | 80% | 16% | 613 | 808 |
| 35% | 80% | 28% | 15% | 80% | 12% | 160 | 207 |
| 35% | 80% | 28% | 18% | 80% | 14.4% | 228 | 297 |
| **35%** | **80%** | **28%** | **20%** | **80%** | **16%** | **297** | **389** |
| 35% | 90% | 31.5% | 18% | 90% | 16.2% | 195 | 256 |
| 35% | 90% | 31.5% | 20% | 90% | 18% | 256 | 334 |
| 38% | 80% | 30.4% | 15% | 80% | 12% | 126 | 164 |
| 38% | 80% | 30.4% | 18% | 80% | 14.4% | 172 | 225 |
| 38% | 80% | 30.4% | 20% | 80% | 16% | 216 | 283 |
| 38% | 80% | 30.4% | 20% | 70% | 14% | 163 | 212 |
| 38% | 80% | 30.4% | 20% | 60% | 12% | 126 | 164 |

^a^ Among those who initiate treatment; ^b^ Among those who are eligible for treatment

|  |
| --- |

**Table 2: Power of Test and Effect Size by Follow-up Time for Reinfection**

| **Follow-up Time Period** | **Re-infection**  **Rate**  **Reduction** | **Power (1-β)** | | | **Total Sample Size Required** | | |
| --- | --- | --- | --- | --- | --- | --- | --- |
| 6 months | 50% | 70% | | | 480 | | |
|  |  |  | 80% |  |  | 624 |  |
| 12 months | 25% | 70% | | | 480 | | |
|  |  | 80% | | | 624 | | |
| 18 months | 20% | 80% | | | 480 | | |
| 24 months | 15% | >85% | | | 480 | | |

*17.3 Describe any procedures that will be used for quality control of collected data.*

Response: OAT site study case managers will review data collected on a case by case basis to ensure all required patient data, lab reports, surveys and questionnaires are completed and documented in patient electronic files. In addition, the data collected during the study will be entered using double data entry by experienced data operators into a database and will be monitored for accuracy and completeness using the data management system. We will conduct continuous data quality checks using the data management system. Participant medical information will be entered into a password protected electronic medical record.

Interview substudy:

The transcripts will be compared against the videoconference or voice recordings for accuracy.

**18.0 Confidentiality**

## A. Confidentiality of Study Data

*Describe the local procedures for maintenance of confidentiality of* ***study data and any records that will be reviewed for data collection****.*

*18.1 A. Where and how will all data and records be stored? Include information about: password protection, encryption, physical controls, authorization of access, and separation of identifiers and data, as applicable. Include physical (e.g. paper)* ***and*** *electronic files.*

Response: We are using My OWN MED (MoM) as the data management system for this protocol. All study data is entered into MoM, which utilizes end-to-end data encryption, SHA384Managed hash algorithm, and password authentication with 24/7/365 network monitoring. All participating institutions have been made aware of MOM data security practices. A copy of the MOM Functional Requirements document is being included in this submission. MoM contains identifiable information. The data can be sent to UB either de-identified or with identifiers. Any database with identifiers will be kept on a secure server accessed by study team members with password protected access. Identifiers will be used as necessary to link billing data to the correct subject, but the data will be deidentified before the analysis stage. Additionally coded information will be stored on a data management server, housed at the Institute for Healthcare Informatics at the University at Buffalo. IHI staff are responsible for routine and continuous monitoring of the network, servers, and overall data security, and our data analytics team also monitors the data for integrity and completeness via data queries and checks. Protections employed by this study will be similar to those that have been enacted in similar clinical research studies. Data will be stored on password protected servers. Passwords will only be available to the study case manager and to the Principal Investigator. Patient identity will not be shared with anyone other than the staff involved in the clinical management of the patient including the physicians who are treating the patients. Patients will be provided a study identification number for all study related data.

Answers from questionnaires and surveys will be entered into MoM. Any paper copies will be kept in locked file cabinets in the clinic where they were done. Billing data will be stored in an excel spreadsheet, under the subject’s study identification number, on password protected computers.

The intake forms containing the SDOH information, or excel sheets that the information from the intake forms has been entered into, or that contain the downloaded PAS data, will be sent to the UB study team either by fax, secure email or through UB Box or secure file transfer. The information will reside in appropriate computer files (i.e., excel, pdfs, cvs) under study IDs, and will be stored on secure servers at Breadcrumb Analytics, or on secure servers at UB including the IHI and CCR. The data needed for biostatistics will be sent to the IHI server and combined with the main study data under study IDs. Any paper copies of the forms will be kept in a locked drawer in a key card protected office in the CTRC. The electronic files will be stored in a password protected file on the S drive.

Interview substudy:

All recordings and transcripts will stored on a password protected secure server.

*18.2 A. How long will the data be stored?*

Response: Data related to the study will be stored for three years after study completion.

All identifiable data in the billing data will be destroyed at the end of the study, defined as the successful publication of a manuscript.

The intake forms (both paper and electronic) and excel sheets that contain the SDOH data will be destroyed/ deleted upon successful publication of that portion of the study.

Interview substudy:

Recordings and complete transcripts will be kept until successful publication. After publication, transcripts will be de-identified and stored indefinitely for future research, and recordings will be deleted.

*18.3 A. Who will have access to the data?*

Response: Because HCV treatment will be a part of the subject’s routine care, identifiable data regarding individual subjects will be available to study staff and providers directly involved in the clinical management of the patients enrolled in the study. Subject data will be de-identified and assigned a study identification number prior to transmission of data for study analysis. Aggregate data will be shared with stakeholders, patient- partners and patient advocates as well as with local IRBs, as appropriate.

The non-coded SDOH information (in the form of the intake forms or downloaded PAS data) will only be accessed by the PI and the study team member(s) at the study site doing the data entry or the downloading and coding of the PAS data. The information will never be in the database in a non-coded form. The coded SDOH data will be combined with the coded data in the main study database for analysis by the biostats team listed on the study. No one who is not currently part of the study team will see the coded database and have access to the code key or identifiable data.

Interview substudy:

Only the study staff at UB will have access to the data.

*18.4 A. Who is responsible for receipt or transmission of the data?*

Response: Ultimately, the principal investigator is responsible for receipt and transmission of all samples and data. Clinical data will be transmitted over web-secured, HIPAA-compliant data management system (MoM) to the Principal investigator and biostatistician responsible for this project. Additionally, this coded information will be stored on a data management server, housed at the Institute for Healthcare Informatics at the University at Buffalo and monitored by study staff at UB Research for the sake of generating reports to identify incomplete or missing data within the MoM system.

The intake forms that contain SDOH data, or the excel sheets that contain the intake forms and PAS data, will be transmitted by fax or electronically. Once entered into the database built for this purpose on the Breadcrumb Analytics server, the data will be securely transferred to the server in IHI, where they can be accessed by the biostatisticians.

Interview substudy:

The study coordinator and PI will be responsible for receipt, The study team member doing the interviews will be responsible for transmission.

*18.5 A. How will the data be transported?*

Response: Data will be transported via electronic documentation to web secure, password protected servers through MoM, a clinical trial management system that will enable us to perform clinic randomization electronically and data collection and transfer from the 12 participating sites to UB research staff for data analysis. All sites enter data into MyOwnMed and the aggregate data is only accessible to UB study staff. Sites do not share their data with any other site, just Dr. Talal and the UB study staff. MOM will also provide consulting and technical support on regulatory and compliance issues related to data sharing throughout the study and will offer resources, such as business associate agreements to permit data sharing between the system and each clinic partner. Data Use Agreements are in place.

SDOH data will be sent via fax or electronically through secure email or Box.

Interview substudy:

All data will be transported electronically.

## B. Confidentiality of Study Specimens

*Describe the local procedures for maintenance of confidentiality of* ***study specimens****.*

☐ **N/A:** No specimens will be collected or analyzed in this research.

*(Skip to Section 19.0)*

*18.1B. Where and how will all specimens be stored? Include information about: physical controls, authorization of access, and labeling of specimens, as applicable.*

Response: The primary purpose of this study is to compare efficacy of HCV treatment between traditional referral and telemedicine. Therefore, handling of specimens will be managed through each individual OAT site with proper labeling of specimens. Patients will be provided a study identification number for all study related data and specimens. Processing, reporting and storing of Standard of Care specimens will be dictated by the commercial vendor laboratory standards of practice. We will also collect 4 vials of blood (60ml) for viral diversity among patients who relapse after treatment and subject to future research. (See Sec.8) These coded specimens will be sent to the biorepository at UB and will be maintained indefinitely. All samples will be labeled with the participant’s study number.

*18.2 B. How long will the specimens be stored?*

Response: specimens for biorepository (if the subject agreed on the ICF to have additional blood drawn for the biorepository) will be stored indefinitely in a biorepository located at 875 Ellicott St Buffalo, N.Y. 14203. A 2ml de-identified aliquot maybe stored at Abbott laboratories in Abbott Park, IL. for possible genome sequencing in the future.

*18.3 B. Who will have access to the specimens?*

*Response:* For specimens housed at the UB biorepository, the PI and study staff associated with the biorepository will have access. Samples sent to commercial vendor laboratories for Standard of Care laboratory assessments will be accessed by employees of these laboratories and will not be used or accessed for future research purposes.

**The following is a description of a possible substudy to happen in the future if scientific questions merit. A new protocol would be submitted for approval if this were to happen.** A request to Abbott Laboratories is for a sub-study to that funded through PCORI. Although patient follow up for two years post treatment cessation was part of the requirements for the PCORI award, no additional funding was provided to enable us to collect and sequence viral isolates. The assumption was that the HCV RNA measurements would be completed by clinical laboratories routinely utilized by each clinic. However, we are interested in increasing the scientific value of the project through the fulfillment of the following specific aims:

1. To assess genetic differences in viral isolates between those patients who have viral recurrence.
2. To identify baseline relatedness between HCV isolates obtained from patients on OAT from different parts of New York State.
3. To identify “hot spots” of viral transmission among PWSUD.
4. To conduct new virus discovery to assess for novel isolates among symptomatic OAT patients from throughout NYS.

Aims related to HCV core antigen

1. Cross-sectional assessment of relationship between HCV core antigen (HCVcAg) and HCV RNA at baseline among OAT patients.
2. Longitudinal assessment of HCVcAg declines in a cohort of PWID on DAA-based therapy.
3. Decline in HCV core antigen for up to 2 years post-treatment completion in a cohort of PWSUD.

1) HCV RNA and HCV genotyping will be performed on the M2000 at Kaleida’s Flint Road facility in a GCP lab.

Whole genome sequencing will be performed at Abbott Laboratories in Abbott Park, IL.

|  |
| --- |

*18.4B. Who is responsible for receipt or transmission of the specimens?*

| Response: Individual sites will be responsible for shipping of specimens according to commercial lab. A lab manual with standard operational procedure and shipping supplies will be provided to each individual clinic for biorepository specimens (sect.16.6). Specimens will be delivered to:  Biorepository- Dr. Andrew Talal  875 Ellicott St 6^th^ fl. Rm.6047  Buffalo, N.Y. 14203 Via FedEx.  Staff at the University of Buffalo with prior IRB authorization to maintain specimens in the Biorepository, will ship via courier from this repository, a de-identified specimen to the Kaleida lab at Flint Rd. Amherst, N.Y. where the Abbott Lab M2000 is |
| --- |

housed, for HCV RNA and genotyping, and a 2 ml aliquot of de-identified blood product will be sent to Abbott Laboratories in Abbott Park, IL. for genome sequencing via shipping supplies provided by Abbott Lab.

*18.5B. How will the specimens be transported?*

Response: In the case of shipment of specimens to the UB biorepository, ambient specimens will be shipped daily to the biorepository using the designated courier (FedEx). Staff at the University of Buffalo with prior IRB authorization to specimens in the Biorepository, will ship via courier from this repository, a deidentified specimen to the Kaleida lab at Flint Rd. Amherst, N.Y. where the Abbott Lab M2000 is housed, for HCV RNA and genotyping, and a 2 ml aliquot of de-identified blood product will be sent to Abbott Laboratories in Abbott Park, IL. for genome sequencing via shipping supplies provided by Abbott Lab.

Standard of Care samples sent to commercial laboratories will be sent using the standard procedure identified by each laboratory at each site.

## 19.0 Provisions to Monitor the Data to Ensure the Safety of Subjects

☐ **N/A:** This study is not enrolling subjects, or is limited to records review procedures only. This section does not apply.

***NOTE: Minimal risk studies may be required to monitor subject safety if the research procedures include procedures that present unique risks to subjects that require monitoring. Some examples include: exercising to exertion, or instruments that elicit suicidality or substance abuse behavior. In such cases, N/A is not an acceptable response.***

*19.1 Describe the plan to periodically evaluate the data collected regarding both harms and benefits to determine whether subjects remain safe.*

Response: During the study start–up phase, a Data Safety Monitoring Board

(DSMB) will be established and meet at a minimum annually. The DSMB will include experts in liver disease, infectious diseases, epidemiology, biostatistics, bioethics and a patient representative, all of whom are independent of the study and institutions. The DSMB will meet initially to review the study aims and protocol, to select variables and procedures for monitoring, and to establish early stopping rules in the case of subject harm. Subsequently, the DSMB will have the opportunity to review trial outcome data at scheduled interim analyses to be conducted in months 24, 36, 48 and 60. Similarly, the IRB at each of the participating institutions could request review of the study in case of concern for patient safety. Rules for early study termination will be established prior to participant engagement.

*19.2 Describe what data are reviewed, including safety data, untoward events, and efficacy data.*

Response: Data to be reviewed will be that involved with direct patient care management by the liver specialists and physician extenders. Data review will include lab values obtained during the clinical management of HCV infection, such as hemoglobin, chemistries, renal and liver function tests as well as HCV RNA. Also, day to day communications with the patient to address patient’s concerns and side effects of treatment will be reviewed on a regular basis by the study team at the participating OAT sites.

*19.3 Describe any safety endpoints.*

Response: Any evidence of serious adverse events and/or death related to telemedicine.

*19.4 Describe how the safety information will be collected (e.g., with case report forms, at study visits, by telephone calls with participants).*

Response: Safety information will be collected by day to day communication with the patient, completion of patient satisfaction surveys, and review of lab reports.

*19.5 Describe the frequency of safety data collection.*

Response: Subjects on methadone maintenance must report to clinics at a frequency that ranges from once daily to once weekly for dosing of methadone. Therefore, any patient concerns or safety laboratory signals may be addressed quickly with the patient by study staff and/or clinical personnel at the OAT clinic.

*19.6 Describe who will review the safety data.*

Response: Data on the study and particularly any safety concerns will be reviewed and discussed by study and clinic staff including the PI as appropriate. Similarly, the IRB at each of the participating clinics and of the participating institutions may request review of the study in case of concern for patient safety. The DSMB will review data annually at a minimum.

*19.7Describe the frequency or periodicity of review of cumulative safety data.*

Response: Cumulative safety data will be reviewed at monthly meetings of Steering committee and at least annually by the DSMB.

*19.8 Describe the statistical tests for analyzing the safety data to determine whether harm is occurring.*

Response: Providers, nurse practitioner/physician assistants will continuously monitor lab results and patient reports of symptoms for assessment of adverse events, and if necessary use disproportionality measures of analysis to assess for potential harm.

*19.9 Describe any conditions that trigger an immediate suspension of the research.*

Response: Evidence of serious adverse events and death related to telemedicine.

## 20.0 Withdrawal of Subjects

☐ **N/A:** This study is not enrolling subjects. This section does not apply.

*20.1 Describe* ***anticipated*** *circumstances under which subjects may be withdrawn from the research without their consent.*

Response: If, in the opinion of the PI continued participation in the study of any subject might lead to undue harm, the subject may be withdrawn without consent. Three consecutive missed appointments will result in a discussion between the principal investigator at the site and the study participant to determine the reason for the missed appointments. Continuation in the study for the participant will be assessed on a case-by-case basis between the study PI and the site PI. Continued HCV treatment will not be affected by study withdrawal and subject to local institutional policy and procedure. Participants in both arms who do not undergo an HCV evaluation within 5 months of enrolling in the study will be withdrawn and will be considered as treatment failures.Any subject will be discontinued from the study if they are discharged from the clinic or if their HCV medication administration lapses for 1 month or more.

|  |
| --- |

*20.2 Describe any procedures for orderly termination.*

*NOTE: Examples may include return of study drug, exit interview with clinician. Include whether additional follow up is recommended for safety reasons for physical or emotional health.*

Response: Orderly termination will proceed as follows. Sites will contact the patient advising of the potential need to terminate participation in the research protocol. If subject is agreeable, a visit may be scheduled at the site to discuss subjects concerns and or reasons for poor adherence.

*20.3 Describe procedures that will be followed when subjects withdraw from the research, including retention of already collected data, and partial withdrawal from procedures with continued data collection, as applicable.*

Response: Subjects will be informed at the time of informed consent that should they choose to withdraw prematurely from the study, they will be asked to complete a last survey and agree to last blood draw to determine viral load status, subsequently, any new and all data collected to date will be included in study results.

## 21.0 Risks to Subjects

*21.1 List the reasonably foreseeable risks, discomforts, hazards, or inconveniences to the subjects related to their participation in the research. Consider physical, psychological, social, legal, and economic risks. Include a description of the probability, magnitude, duration, and reversibility of the risks.*

*NOTE: Breach of confidentiality is always a risk for identifiable subject data.*

Response: The primary risk potential is breach of participant confidentiality and privacy and injury due to blood drawing. Blood obtained for HCV RNA levels will be drawn by experienced laboratory personnel who have been trained in phlebotomy. Data protections will be designed in collaboration with the information technology departments at each of the participating sites throughout the study to minimalize the risk of breach of confidentiality.

Interview substudy:

Breach of confidentiality is a risk. Subjects may feel uncomfortable with some interview questions.

For the focus groups: While we are not specifically looking for information that may be damaging, we are asking about experiences from the staff and administrators’ places of employment, and due to the free form nature of the focus groups, participants may offer opinions and information that may be damaging to their employment and/or reputation. Due to the nature of the focus groups, multiple people from each clinic may be present, as well as other professionals from other clinics, and therefore participants would not have confidentiality from other participants. No content from the focus groups will be published or shared in an identifiable manner.

*21.2 Describe procedures performed to lessen the probability or magnitude of risks, including procedures being performed to monitor subjects for safety.*

Response: To minimize risk, blood will be drawn in medical settings with protections such as bandages and immediate access to medical personnel in case of severe injury. Discussions of study enrollment will be done in a private setting, between the study case manager and the potential study participant at the individual participating OAT clinic site. Similarly, for patients in the telemedicine arm, HCV therapies will be dispensed at the methadone dispensing window at the same time as methadone to minimize the chance that a person might be observed taking HCV medications. Consent will be obtained to disclose medical information to their medical providers and HCV specialists in order to follow up on referral requests. Participants will also be asked to sign a confidentiality statement acknowledging the importance of not disclosing personal information to other participants. Participant medical information will be entered into a password protected electronic medical record. Data collected during the study will be entered using data entry by experienced data operators into MoM, or a secure database created by Breadcrumb Analytics. This de-identified data will be monitored for accuracy and completeness via reports generated by the Institute for Healthcare Informatics Department at the University at Buffalo

Interview substudy:

Subjects’ names will not be included in recordings or transcripts. All recordings and transcripts will be stored electronically on a password protected server.

*21.3 If applicable, indicate* ***which procedures*** *may have risks to the subjects that are currently unforeseeable.*

Response: While measures will be taken to avoid breach of confidentiality, technology at all levels is at risk for corruption of data servers as well as cyber hacking of information.

*21.4 If applicable, indicate which research procedures may have risks to an embryo or fetus should the subject be or become pregnant.*

Response: N/A

*21.5 If applicable, describe risks to others who are not subjects.*

Response: N/A

## 22.0 Potential Benefits to Subjects

*22.1 Describe the potential benefits that individual subjects may experience by taking part in the research. Include the probability, magnitude, and duration of the potential benefits. Indicate if there is no direct benefit.*

*NOTE: Compensation* ***cannot*** *be stated as a benefit.*

Response: For those participants who follow through with treatment from referral to a liver specialist or via telemedicine intervention, the expected benefit will be sustained virologic response. Additionally, many patients who have resolved HCV report improved quality of life, diminished fatigue, a more positive attitude toward life in general with the knowledge that they have eliminated a chronic viral infection.

## 23.0 Compensation for Research-Related Injury

☐ **N/A:** The research procedures for this study do not present risk of research related injury (e.g. survey studies, records review studies). This section does not apply.

***23.1 If the research procedures carry a risk of research related injury,*** *describe the available compensation to subjects in the event that such injury should occur.*

Response: Since this is a study of telemedicine-based treatment approaches for HCV, we do not anticipate a great deal of risk associated with the protocol.

Therefore, no compensation will be available to subjects in the event of injury. We do acknowledge, however, that we are assessing a treatment delivery modality that will involve the use of FDA-approved medications at indicated doses and durations; these medications may have their own associated adverse events. Although not part of the research, per se, in the event of injury to the study subject, immediate medical attention will be provided at the clinic where they customarily receive treatment or by referral from the study participating clinic to a local hospital.

*23.2 Provide a copy of contract language, if any, relevant to compensation for research related injury.*

*NOTE: If the contract is not yet approved at the time of this submission, submit the current version here. If the contract is later approved with* ***different*** ***language regarding research related injury****, you must modify your response here and submit an amendment to the IRB for review and approval.*

Response: There is no specific contract language from PCORI related to compensation for research related injury.

## 24.0 Economic Burden to Subjects

*24.1 Describe any costs that subjects may be responsible for because of participation in the research.*

*NOTE: Some examples include transportation or parking.*

Response: Inclusion criteria require that all subjects must be covered by medical insurance as is the case for 95% of patients on OAT in New York State. Subjects consenting to participate in either arm will be responsible for any and all copayments identified by medical insurers. We will attempt to have visits for subjects managed by telemedicine scheduled during their regularly scheduled methadone visits. However, there may be some costs related to travel for unscheduled visits. Therefore, we do not anticipate significant added financial burden incurred due to transportation required to travel to the methadone clinic for study-related visits.

☐ **N/A:** This study is not enrolling subjects, or is limited to records review procedures only. This section does not apply.

## 25.0 Compensation for Participation

*25.1 Describe the amount and timing of any compensation to subjects, including monetary, course credit, or gift card compensation.*

Response: Subjects will receive $25 each for completion of consent and blood draw, and $15 for completion of each survey as well questionnaire/survey. Participants in the telemedicine arm will be reimbursed a total of $130 and in the usual care arm a total of $130.

| Item administered | Cost per admini-stration | # of times administered | Total admini-stration cost |
| --- | --- | --- | --- |
| Informed consent and blood draw | $25 | 1 | $25 |
| Sociodemo-graphics | $15 | 1 | $15 |
| Mini mental | $15 | 1 | $15 |
| DAST | $15 | 2 | $30 |
| NIDA Quick Screen | $15 | 1 | $15 |
| HCV patient satisfaction | $15 | 2 | $30 |
| Total |  |  | $130 |

DAST-10, drug abuse screening test; NIDA, National Institute on Drug Abuse; HCV, hepatitis C virus.

Subjects will be compensated with petty cash at the end of each visit and per survey completed.

Subjects who participate in study dissemination interviews and media appearances will be compensated $25 per hour with petty cash at the end of each interview.

If a subject is reinfected and agrees to have additional blood draws, they will receive $25 for the genotyping blood draw visit, and if they begin retreatment, $25 for each blood draw visit, up to 5.

Interview substudy:

PCORI Study subjects and non-participants will be compensated $25 per interview or focus group session. Compensation may be in the form of gift card or cash. It will be available immediately after the interview at the substance use treatment facility for the subject to pick up at their convenience, or mailed in a secure fashion. If possible (depending on union/site rules) staff will be compensated $25 for interviews.

| ☐ | **N/A:** This study is not enrolling subjects, or is limited to records review procedures only. This section does not apply. |
| --- | --- |
| ☐ | **N/A:** There is no compensation for participation. This section does not apply. |

## 26.0 Consent Process

*26.1 Indicate whether you will be obtaining consent.*

*NOTE: This does not refer to consent documentation, but rather whether you will be obtaining permission from subjects to participate in a research study. Consent documentation is addressed in Section 27.0.*

☒ ***Yes*** *(If yes, Provide responses to each question in this Section)*

☐ ***No*** *(If no, Skip to Section 27.0)*

*26.2 Describe where the consent process will take place. Include steps to maximize subjects’ privacy.*

Response: Consenting of subjects will be completed at the individual participating clinic sites by designated study staff. Discussions of study enrollment and consenting will be done in a private setting between the study case manager at the participating OAT clinic and the potential study participant. All participating OAT clinics must provide a patient-friendly, private examination room for discussion of HCV detection, study information, and consenting.

Subjects who are found to be reinfected will be asked to sign an ICF addendum for the collection of additional blood draws.

Interview substudy:

For interviews, the consent process will take place in a private room, or over the phone or videoconference while both parties are in a private room.

*26.3 Describe how you will ensure that subjects are provided with a sufficient period of time to consider taking part in the research study.*

*NOTE: It is always a requirement that a prospective subject is given sufficient time to have their questions answered and consider their participation. See “SOP: Informed Consent Process for Research (HRP-090)” Sections 5.5 and 5.6.*

Response: Patients identified as HCV antibody positive will be given their results and information regarding HCV infection in the form a brochure provided by SAMHSA. At that time, the case study manager will inform the patient of the study and will provide them with a copy of the consent for their review. Potential subjects will be given as much time as required to review the consent, including taking it home with them for consideration of signing at another visit. As patients attend the OAT program several times per week, asking them to return on a subsequent day should not pose an undue burden on patients. All patients will be encouraged to ask questions and to discuss potential enrollment with family members, friends, and other providers. Patients may enroll at any time during the control or intervention time frames.

Interview substudy:

Subjects may take their time deciding whether they would like to participate.

*26.4 Describe any process to ensure ongoing consent, defined as a subject’s willingness to continue participation for the duration of the research study.*

Response: Subjects will be informed of their right to withdraw from the study at any time without risk of jeopardizing ongoing participation in the OAT program.

Interview substudy:

If subjects seem uncomfortable with a question, they will be reminded that they may skip any questions, or stop the interview at any time.

*26.5 Indicate whether you will be following “SOP: Informed Consent Process for Research (HRP-090).” If not, or if there are any exceptions or additional details to what is covered in the SOP, describe:*

- *The role of the individuals listed in the application who are involved in the consent process*
- *The time that will be devoted to the consent discussion*
- *Steps that will be taken to minimize the possibility of coercion or undue influence*
- *Steps that will be taken to ensure the subjects’ understanding*

Response: Subjects who are interested in participating in interviews and speaking engagements during study dissemination activities will sign a separate consent addendum or will certify that they agree to be coauthors on publications resulting from this investigation. These individuals will serve as sub-study participants; they are not methadone patients.

☒ We have reviewed and will be following “SOP: Informed Consent Process for Research (HRP-090).”

***Non-English Speaking Subjects***

☐ **N/A:** This study will not enroll Non-English speaking subjects. *(Skip to Section 26.8)*

*26.6 Indicate which language(s) other than English are likely to be spoken/understood by your prospective study population or their legally authorized representatives.*

*NOTE: The response to this Section should correspond with your response to Section 6.4 of this protocol.*

Response: English and Spanish are the two most common languages spoken by a great majority of methadone maintained patients in New York

State.

*If subjects who do not speak English will be enrolled, describe the process to ensure that the oral and written information provided to those subjects will be in that language. Indicate the language that will be used by those obtaining consent.*

*NOTE: Guidance is provided on “SOP: Informed Consent Process for Research (HRP-090).”*

Response: Study materials (including informed consent) will be available in both English and Spanish, the two languages that are spoken by the great majority of methadone-maintained patients in NYS. In most circumstances, Spanish-speaking clinic staff at participating OAT clinics are available to reach out to non- English speaking patients so that they may be given the opportunity to consider enrollment.

***Cognitively Impaired Adults***

☒ **N/A**: This study will not enroll cognitively impaired adults.

*(Skip to Section 26.9)*

*26.7 Describe the process to determine whether an individual is capable of consent.*

Response: N/A

***Adults Unable to Consent***

☒ **N/A**: This study will not enroll adults unable to consent.

(*Skip to Section 26.13)*

*When a person is not capable of consent due to cognitive impairment, a legally authorized representative should be used to provide consent (Sections 26.9 and 26.10)* ***and, where possible, assent of the individual should also be solicited*** *(Sections 26.11 and 26.12).*

*26.8 Describe how you will identify a Legally Authorized Representative (LAR). Indicate that you have reviewed the “SOP: Legally Authorized Representatives, Children, and Guardians (HRP-013)” for research in New York State.*

*NOTE: Examples of acceptable response includes: verifying the electronic medical record to determine if an LAR is recorded.*

Response: N/A

☐ We have reviewed and will be following “SOP: Legally Authorized

Representatives, Children, and Guardians (HRP-013).”

*26.9* ***For research conducted outside of New York State****, provide information that describes which individuals are authorized under applicable law to consent on behalf of a prospective subject to their participation in the research. One method of obtaining this information is to have a legal counsel or authority review your protocol along with the definition of “legally authorized representative” in “SOP: Legally Authorized Representatives, Children, and Guardians (HRP-013).”*

Response: This study will be conducted only in New York State.

*26.10 Describe the process for* ***assent of the*** ***adults****:*

- *Indicate whether assent will be obtained from all, some, or none of the subjects. If some, indicate which adults will be required to assent and which will not.*

Response:

- *If assent will not be obtained from some or all subjects, provide an explanation of why not.*

Response: N/A

*26.11 Describe whether* ***assent of the*** ***adult*** *subjects will be documented and the process to document assent.*

*NOTE: The IRB allows the person obtaining assent to document assent on the consent document using the “Template Consent Document (HRP-502)” Signature Block for Assent of Adults who are Legally Unable to Consent.*

Response:

***Subjects who are not yet Adults (Infants, Children, and Teenagers)***

☒ **N/A**: This study will not enroll subjects who are not yet adults. *(Skip to Section 27.0)*

*26.12Describe the criteria that will be used to determine* ***whether a prospective subject has not attained the legal age for consent to treatments or procedures involved in the research*** *under the applicable law of the jurisdiction in which the research will be conducted* ***(e.g., individuals under the age of 18 years)****. For research conducted in NYS, review “SOP: Legally Authorized Representatives, Children, and Guardians (HRP-013)” to be aware of which individuals in the state meet the definition of “children.”*

*NOTE: Examples of acceptable responses include: verification via electronic medical record, driver’s license or state-issued ID, screening questionnaire.*

Response: Verification of a subject’s age will be achieved via electronic medical records and/or photo ID.

***26.13 For research conducted outside of New York State****, provide information that describes which persons have not attained the legal age for consent to treatments or procedures involved the research, under tctedhe applicable law of the jurisdiction in which research will be conducted. One method of obtaining this information is to have a legal counsel or authority review your protocol*

*along the definition of “children” in “SOP: Legally Authorized Representatives, Children, and Guardians (HRP-013).”*

Response: This study will be conducted entirely in NYS.

*26.14 Describe whether parental permission will be obtained from:*

Response: there will not be children under the age of 18 enrolled in this study.

☐ One parent even if the other parent is alive, known, competent, reasonably available, and shares legal responsibility for the care and custody of the child.

☐ Both parents unless one parent is deceased, unknown, incompetent, or not reasonably available, or when only one parent has legal responsibility for the care and custody of the child.

☐ Parent permission will not be obtained. A waiver of parent permission is being requested.

*NOTE: The requirement for parent permission is a protocol-specific determination made by the IRB based on the risk level of the research. For guidance, review the*

*“CHECKLIST: Children (HRP-416).”*

*26.15Describe whether permission will be obtained from individuals* ***other than parents****, and if so, who will be allowed to provide permission. Describe your procedure for determining an individual’s authority to consent to the child’s general medical care.*

Response: No subjects under the age of 18 years of age will be enrolled in this study.

*26.16Indicate whether assent will be obtained from all, some, or none of the* ***children****. If assent will be obtained from some children, indicate which children will be required to assent.*

Response: N/A

*26.17 When assent of children is obtained, describe how it will be documented.*

Response: N/A

## 27.0 Waiver or Alteration of Consent Process

***Consent will not be obtained, required information will not be disclosed, or the research involves deception.***

☐ **N/A:** A waiver or alteration of consent is not being requested.

*27.1 If the research involves a waiver or alteration of the consent process, please review the “CHECKLIST: Waiver or Alteration of Consent Process (HRP-410)” to ensure that you have provided sufficient information for the IRB to make the determination that a waiver or alteration can be granted.*

*NOTE: For records review studies, the first set of criteria on the “CHECKLIST: Waiver or Alteration of Consent Process (HRP-410)” applies.*

Response: (Modification 2/27/2020) We are requesting a waiver of consent only for the analysis of the subjects’ financial data for those in the telemedicine arm and the subset of usual care arm subjects who were treated by the PI. The workforce and resource analysis was not planned at the beginning of the study, so permission to collect billing data was not included in the consent. At this point in the timeline of the study, it is impracticable to re-consent everyone that the PI treated. Approximately 5% of the subjects are deceased or lost to follow up, and many of the subjects are in long term follow up, with study visits only every 6 months. Given the nature of the study population, with approximately 15% discontinuation from the methadone treatment program annually, the rate of subjects who will be lost to follow up during the long term follow up period is expected to be higher than the average patient population.

The major point is that long term follow up lasts for two years. With patients who have discontinued from the study because they no longer have follow up, as well as those who have transferred, expired or been imprisoned, approximately 30% of telemedicine patients are no longer in contact with the study team.

As the provider of the telemedicine or usual care, the PI and his practice plan (UBMD) are already in possession of the data for non-research purposes, so no further patient records would have to be accessed to gather the data. In addition, the use of these data would not require any patient interactions.

In order to properly analyze socioeconomic variable differences by region and cost benefit measures by liver disease stages, we would like to include the entire telemedicine population and the few usual care subjects treated by the PI, and not just the smaller subset of those from whom we can get consent from at this point.

(Modification 5/8/2020) Based on the initial analysis of the billing data that was collected in our previous modification, we are asking for a waiver of consent to try to collect the retrospective billing data for the remainder of the usual care arm subjects who were not treated by the PI. This study is a comparison of telemedicine vs usual care, and usual care in the vast majority of cases is offsite referral. We would request the billing data from the subject’s insurance company, or secondarily, from their treating physician or clinic.

We are also asking for a waiver of consent to retrospectively collect data points from the subjects’ medical records at the substance use treatment facility and OASAS’s servers to analyze their social determinants of health (SDOH). SDOH are behavioral, environmental, and demographic characteristics that can affect a patient’s health. Upon admission to a substance use treatment facility, all patients are requested to complete a form that originates from the New York State Office of Addiction Services and Supports (OASAS) and collects a variety of psychosocial and economic information. A copy of the form from the 01 clinic, a powerpoint showing the site 11 clinic, and the PAS-44 and PAS26 have been added to Supporting Documents and all clinics in NYS collect similar information as part of their admission process. The clinics have their own forms, and then the clinic fills out the PAS-44 and PAS-26 for OASAS. The original award that funds the study was recently amended by PCORI to include this analysis.

|  |  |
| --- | --- |
|  | |

*27.2 If the research involves a waiver of the consent process for planned emergency research, please review the “CHECKLIST: Waiver of Consent for Emergency Research (HRP-419)” to ensure you have provided sufficient information for the IRB to make these determinations. Provide any additional information necessary here:*

Response: N/A

## 28.0 Process to Document Consent

☐ **N/A:** A Waiver of Consent is being requested.

*(Skip to Section 29.0)*

*28.1Indicate whether you will be following “SOP: Written Documentation of Consent (HRP-091).” If not or if there are any exceptions, describe whether and how consent of the subject will be obtained including whether or not it will be documented in writing.*

*NOTE: If your research presents no more than minimal risk of harm to subjects and involves no procedures for which written documentation of consent is normally required outside of the research context, the IRB will generally waive the requirement to obtain written documentation of consent. This is sometimes referred to as ‘verbal consent.’ Review “CHECKLIST: Waiver of Written Documentation of Consent (HRP-411)” to ensure that you have provided sufficient information.*


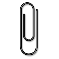
*If you will document consent in writing, attach a consent document with your submission. You may use “TEMPLATE CONSENT DOCUMENT (HRP-*

*502)”. If you will obtain consent, but not document consent in writing, attach the script of the information to be provided orally or in writing (i.e. consent script or Information Sheet).*

Response:

Interview substudy:

If the subject is consented in person, a written, signed consent form will be available. If the subject is consented remotely, either over the phone or over videoconference, the person obtaining consent will document the subject’s verbal consent. A hard copy of the consent will be left in participant’s OTP for them to pick up or emailed/mailed to them in a secure manner. No matter which of these methods are used, consent to be recorded for interviews and/or focus groups will be reitereated at the beginning of the activity and prior to recording.

☐ We will be following “SOP: Written Documentation of Consent” (HRP-091).

## 29.0 Multi-Site Research (Multisite/Multicenter Only)

☐ **N/A:** This study is not an investigator-initiated multi-site study. This section does not apply.

*29.1 If this is a multi-site study* ***where you are the lead investigator****, describe the processes to ensure communication among sites, such as:*

- All sites have the most current version of the IRB documents, including the protocol, consent document, and HIPAA authorization.
- All required approvals have been obtained at each site (including approval by the site’s IRB of record).
- All modifications have been communicated to sites, and approved (including approval by the site’s IRB of record) before the modification is implemented.
- All engaged participating sites will safeguard data as required by local information security policies.
- *All local site investigators conduct the study appropriately.*
- *All non-compliance with the study protocol or applicable requirements will be reported in accordance with local policy.*

Response:

- As the lead site, the UB Research Center will ensure that once an IRB amendment is approved by the UB IRB, the revised protocol, consents, and HIPAA authorization (if applicable) is distributed to all of the study sites.
- As the lead-site, we will maintain documentation that all required approvals will have been obtained at each site prior to implementation of any study related procedures, including approval by the site’s IRB of record.
- As the lead site, we will ensure that all modifications have been communicated to sites and approved (including approval by the site’s IRB of record) before the modification is implemented.

| • | As the lead site we will ensure all engaged participating sites will safeguard data as required by local information security policies. |  |
| --- | --- | --- |
| • | As the lead site we will ensure all local site investigators conduct the study appropriately. |  |
| • | All non-compliance with the study protocol or applicable requirements will be reported in accordance with local policy. | |

*29.2 Describe the method for communicating to engaged participating sites:*

- *Problems*
- *Interim results*
- *Study closure*

Response: Communication to engaged participating sites will be achieved through a Governance Plan. A management structure will be organized through a Steering committee and four standing committees.

The Governance Plan consists of:

Steering committee: will set priorities, oversee timely completion of tasks and monitor the overall budget for the study. They will also supplement the Data and Safety Monitoring Plan and will review project data in that capacity as needed.

Implementation Committee: This committee will oversee intervention activities, monitor quality of interventions, and make adjustments as needed.

Evaluation Committee: Will oversee all aspects related to data management and analysis, including the preparation of study instruments and development of the evaluation tool for capturing data from the electronic chart reviews.

Patient Advisory Committee: Will review procedures to permit patients an opportunity to discuss their concerns, and to maintain the patient centeredness of the study. All case managers will attend the regular meeting with the PAC at each clinic site to ensure timely resolution of patient-centered ongoing concerns or obstacles to study recruitment.

Sustainability Committee: will include all stakeholders and will meet annually to review study progress and to provide advice on sustainability.

|  |
| --- |

*29.3 Indicate the total number of subjects that will be enrolled or records that will be reviewed across all sites.*

Response: The total sample size is projected to be 624 patients enrolled from 12 participating OAT programs throughout New York State.

*29.4If this is a multicenter study for which UB will serve as the IRB of record, and subjects will be recruited by methods not under the control of the local site (e.g., call centers, national advertisements) describe those methods.*

Response: We will seek to engage a local IRB at each individual site for review and approval of the study protocol. In the case that a site does not have a local IRB, University at Buffalo IRB will be acting as the IRB of record.

## 30.0 Banking Data or Specimens for Future Use

☐ **N/A:** This study is not banking data or specimens for future use or research outside the scope of the present protocol. This section does not apply.

*30.1 If data or specimens will be banked (stored) for* ***future use, that is, use or research outside of the scope of the present protocol****, describe where the data/specimens will be stored, how long they will be stored, how the data/specimens will be accessed, and who will have access to the data/specimens.*

*NOTE: Your response here must be consistent with your response at the “What happens if I say yes, I want to be in this research?” Section of the Template Consent Document (HRP-502).*

Response: A separate check box will be included in the informed consent to enable the collection of blood specimens at the time of enrollment (baseline), during treatment and up to two years after treatment for storage in the biorepository for viral genetic testing. Patients will be given a copy of the consent and will be counselled that their participation in this aspect of the study is voluntary and will not disqualify their enrollment should they choose not to participate in donating a serum specimen. Similarly, should they choose to request that their sample be discarded at any time, study personnel will adhere to their request. Samples will be stored indefinitely and only study personnel at UB will have access to them. From this repository a de-identified specimen will be sent to the Kaleida lab at Flint Rd. Amherst, N.Y. where the Abbott Lab M2000 is housed for HCV RNA and genotyping. A 2ml de-identified aliquot will be stored at Abbott laboratories in Abbott Park, IL. for genome sequencing.

Interview transcripts will be de-identified and banked indefinitely for future research purposes.

*30.2 List the data to be stored or associated with each specimen.*

Response: Medical information captured within the MoM system will be transferred from the subject’s electronic medical record to our database by secure transfer from MoM to University at Buffalo’s secure Data base housed at the Institute for Healthcare Informatics. The specimen will be deidentified and assigned a sequential number and site code identifying each specimen. The data to be collected are as follows: Gender, age at start of study, Diagnosis, Co-morbidities, smoking history, height, weight, fasting status, social history including drug and alcohol use and frequency, race, geographical location of residence and primary language.

|  |
| --- |

*30.3 Describe the procedures to release banked data or specimens for future uses, including: the process to request a release, approvals required for release, who can obtain data or specimens, and the data to be provided with specimens.*

*Response:* Storage and release of the specimens will be under the auspices of the PI,

Andrew H. Talal, MD and based upon submission of a written request to Dr. Andrew Talal, MD, 875 Ellicott St., Buffalo, NY 14203, and an IRB approved protocol. The specimens and associated data (stated in 28.2) will then be released by the director of the biorepository. The specimens will be packaged and shipped on dry ice to the designated recipient via chain of custody. A de-identified specimen will be sent via courier to the

Kaleida lab at Flint Rd. Amherst, N.Y. where the Abbott Lab M2000 is housed for HCV RNA and genotyping. A 2ml de-identified aliquot will be stored at Abbott laboratories in Abbott Park, IL. for genome sequencing using shipping supplies provided by Abbott labs.

All future use of data from this study outside the scope of this protocol will be submitted to the IRB for review.

|  |
| --- |

## 31.0 Drugs or Devices

☒ **N/A:** This study does not involve drugs or devices. This section does not apply.

*31.1 If the research involves drugs or devices, list and describe all drugs and devices used in the research, the purpose of their use, and their regulatory approval status.*

Response: N/A. We will be using FDA approved medications for HCV treatment consistent with standard approval.

*31.2 Describe your plans to store, handle, and administer those drugs or devices so that they will be used only on subjects and be used only by authorized investigators.*

Response: N/A. All drugs patients receive are standard of care, not part of the research study. All medications will be stored according to the manufacturer’s recommendations, and distributed to subjects enrolled in the study, along with dosing of methadone by authorized personnel.

***If the drug is investigational (has an IND) or the device has an IDE or a claim of abbreviated IDE (non-significant risk device), include the following information:***

*31.3 Identify the holder of the IND/IDE/Abbreviated IDE.*

Response: N/A. No investigational drugs will be utilized in this study. All drugs used for HCV treatment in this study are FDA approved used as Standard of Care.

*31.4 Explain procedures followed to comply with FDA sponsor requirements for the following:*

|  |  | ***Applicable to:*** |  |
| --- | --- | --- | --- |
| ***FDA Regulation*** | ***IND Studies*** | ***IDE studies*** | ***Abbreviated IDE studies*** |
| ***21 CFR 11*** | ***X*** | ***X*** |  |
| ***21 CFR 54*** | ***X*** | ***X*** |  |
| ***21 CFR 210*** | ***X*** |  |  |
| ***21 CFR 211*** | ***X*** |  |  |
| ***21 CFR 312*** | ***X*** |  |  |
| ***21 CFR 812*** |  | ***X*** | ***X*** |
| ***21 CFR 820*** |  | ***X*** |  |

Response: This study will only use FDA approved medications for HCV treatment.

## 32.0 Humanitarian Use Devices

☒ **N/A:** This study does not involve humanitarian use devices. This does not apply.

*32.1 For Humanitarian Use Device (HUD) uses provide a description of the device, a summary of how you propose to use the device, including a description of any screening procedures, the HUD procedure, and any patient follow-up visits, tests or procedures.*

Response:

*32.2 For HUD uses provide a description of how the patient will be informed of the potential risks and benefits of the HUD and any procedures associated with its use.*

Response: N/A
